# Supplementary material for: Overlapping cell population expression profiling and regulatory inference in C. elegans
Source: BMC Genomics. 2016 Feb 29;17:159. doi: 10.1186/s12864-016-2482-z (PMC4772325; doi:10.1186/s12864-016-2482-z)
Supplement: Additional file 13: — Web supplement. (DOC 21 kb) [file 12864_2016_2482_MOESM13_ESM.zip › sortWeb/clusters/hier.300.clusters/46.html]

Cluster 46 

## Cluster 46

### Expression

| cnd-1 rep. 1 | cnd-1 rep. 2 | cnd-1 rep. 3 | pha-4 rep. 1 | pha-4 rep. 2 | pha-4 rep. 3 | ceh-27 | ceh-36 | ceh-6 | F21D5.9 | mir-57 | mls-2 | pal-1 | pros-1 | ttx-3 | unc-130 | hlh-16 | irx-1 | ceh-6 (+) hlh-16 (+) | ceh-6 (+) hlh-16 (-) | ceh-6 (-) hlh-16 (+) | cnd-1 singlets | pha-4 singlets | 0 | 60 | 120 | 150 | 180 | 240 | 330 | 390 | 420 | 480 | 540 | 570 | 600 | 630 | 660 | NAME | Functional description |
| --- | --- | --- | --- | --- | --- | --- | --- | --- | --- | --- | --- | --- | --- | --- | --- | --- | --- | --- | --- | --- | --- | --- | --- | --- | --- | --- | --- | --- | --- | --- | --- | --- | --- | --- | --- | --- | --- | --- | --- |
|  |  |  |  |  |  |  |  |  |  |  |  |  |  |  |  |  |  |  |  |  |  |  |  |  |  |  |  |  |  |  |  |  |  |  |  |  |  | *dod-23* | Downstream Of DAF-16 (regulated by DAF-16) |
|  |  |  |  |  |  |  |  |  |  |  |  |  |  |  |  |  |  |  |  |  |  |  |  |  |  |  |  |  |  |  |  |  |  |  |  |  |  | C48B4.10 |  |
|  |  |  |  |  |  |  |  |  |  |  |  |  |  |  |  |  |  |  |  |  |  |  |  |  |  |  |  |  |  |  |  |  |  |  |  |  |  | *cutl-6* | CUTiclin-Like |
|  |  |  |  |  |  |  |  |  |  |  |  |  |  |  |  |  |  |  |  |  |  |  |  |  |  |  |  |  |  |  |  |  |  |  |  |  |  | C08F8.6 |  |
|  |  |  |  |  |  |  |  |  |  |  |  |  |  |  |  |  |  |  |  |  |  |  |  |  |  |  |  |  |  |  |  |  |  |  |  |  |  | *ugt-65* | UDP-GlucuronosylTransferase |
|  |  |  |  |  |  |  |  |  |  |  |  |  |  |  |  |  |  |  |  |  |  |  |  |  |  |  |  |  |  |  |  |  |  |  |  |  |  | T18D3.5 |  |
|  |  |  |  |  |  |  |  |  |  |  |  |  |  |  |  |  |  |  |  |  |  |  |  |  |  |  |  |  |  |  |  |  |  |  |  |  |  | F22D6.2 |  |
|  |  |  |  |  |  |  |  |  |  |  |  |  |  |  |  |  |  |  |  |  |  |  |  |  |  |  |  |  |  |  |  |  |  |  |  |  |  | F54E4.4 |  |
|  |  |  |  |  |  |  |  |  |  |  |  |  |  |  |  |  |  |  |  |  |  |  |  |  |  |  |  |  |  |  |  |  |  |  |  |  |  | T20F5.4 |  |
|  |  |  |  |  |  |  |  |  |  |  |  |  |  |  |  |  |  |  |  |  |  |  |  |  |  |  |  |  |  |  |  |  |  |  |  |  |  | H03E18.1 |  |
|  |  |  |  |  |  |  |  |  |  |  |  |  |  |  |  |  |  |  |  |  |  |  |  |  |  |  |  |  |  |  |  |  |  |  |  |  |  | *abu-11* | Activated in Blocked Unfolded protein response |
|  |  |  |  |  |  |  |  |  |  |  |  |  |  |  |  |  |  |  |  |  |  |  |  |  |  |  |  |  |  |  |  |  |  |  |  |  |  | F41E6.11 |  |
|  |  |  |  |  |  |  |  |  |  |  |  |  |  |  |  |  |  |  |  |  |  |  |  |  |  |  |  |  |  |  |  |  |  |  |  |  |  | C54D2.1 |  |
|  |  |  |  |  |  |  |  |  |  |  |  |  |  |  |  |  |  |  |  |  |  |  |  |  |  |  |  |  |  |  |  |  |  |  |  |  |  | F22E5.1 |  |
|  |  |  |  |  |  |  |  |  |  |  |  |  |  |  |  |  |  |  |  |  |  |  |  |  |  |  |  |  |  |  |  |  |  |  |  |  |  | K08F9.5 |  |
|  |  |  |  |  |  |  |  |  |  |  |  |  |  |  |  |  |  |  |  |  |  |  |  |  |  |  |  |  |  |  |  |  |  |  |  |  |  | K08F9.3 |  |
|  |  |  |  |  |  |  |  |  |  |  |  |  |  |  |  |  |  |  |  |  |  |  |  |  |  |  |  |  |  |  |  |  |  |  |  |  |  | *gst-3* | Glutathione S-Transferase |
|  |  |  |  |  |  |  |  |  |  |  |  |  |  |  |  |  |  |  |  |  |  |  |  |  |  |  |  |  |  |  |  |  |  |  |  |  |  | *nhr-65* | Nuclear Hormone Receptor family |
|  |  |  |  |  |  |  |  |  |  |  |  |  |  |  |  |  |  |  |  |  |  |  |  |  |  |  |  |  |  |  |  |  |  |  |  |  |  | W05H5.1 |  |
|  |  |  |  |  |  |  |  |  |  |  |  |  |  |  |  |  |  |  |  |  |  |  |  |  |  |  |  |  |  |  |  |  |  |  |  |  |  | K04G2.12 |  |
|  |  |  |  |  |  |  |  |  |  |  |  |  |  |  |  |  |  |  |  |  |  |  |  |  |  |  |  |  |  |  |  |  |  |  |  |  |  | K10D11.7 |  |
|  |  |  |  |  |  |  |  |  |  |  |  |  |  |  |  |  |  |  |  |  |  |  |  |  |  |  |  |  |  |  |  |  |  |  |  |  |  | F20B10.3 |  |
|  |  |  |  |  |  |  |  |  |  |  |  |  |  |  |  |  |  |  |  |  |  |  |  |  |  |  |  |  |  |  |  |  |  |  |  |  |  | R05H5.7 |  |
|  |  |  |  |  |  |  |  |  |  |  |  |  |  |  |  |  |  |  |  |  |  |  |  |  |  |  |  |  |  |  |  |  |  |  |  |  |  | *aat-6* | Amino Acid Transporter |
|  |  |  |  |  |  |  |  |  |  |  |  |  |  |  |  |  |  |  |  |  |  |  |  |  |  |  |  |  |  |  |  |  |  |  |  |  |  | R01E6.2 |  |
|  |  |  |  |  |  |  |  |  |  |  |  |  |  |  |  |  |  |  |  |  |  |  |  |  |  |  |  |  |  |  |  |  |  |  |  |  |  | *nmur-4* | NMUR (NeuroMedin U Receptor) homolog |
|  |  |  |  |  |  |  |  |  |  |  |  |  |  |  |  |  |  |  |  |  |  |  |  |  |  |  |  |  |  |  |  |  |  |  |  |  |  | *nhr-211* | Nuclear Hormone Receptor family |
|  |  |  |  |  |  |  |  |  |  |  |  |  |  |  |  |  |  |  |  |  |  |  |  |  |  |  |  |  |  |  |  |  |  |  |  |  |  | ZC21.3 |  |
|  |  |  |  |  |  |  |  |  |  |  |  |  |  |  |  |  |  |  |  |  |  |  |  |  |  |  |  |  |  |  |  |  |  |  |  |  |  | ZC21.9 |  |
|  |  |  |  |  |  |  |  |  |  |  |  |  |  |  |  |  |  |  |  |  |  |  |  |  |  |  |  |  |  |  |  |  |  |  |  |  |  | C09G9.5 |  |
|  |  |  |  |  |  |  |  |  |  |  |  |  |  |  |  |  |  |  |  |  |  |  |  |  |  |  |  |  |  |  |  |  |  |  |  |  |  | F13B6.3 |  |
|  |  |  |  |  |  |  |  |  |  |  |  |  |  |  |  |  |  |  |  |  |  |  |  |  |  |  |  |  |  |  |  |  |  |  |  |  |  | *nhr-37* | Nuclear Hormone Receptor family |
|  |  |  |  |  |  |  |  |  |  |  |  |  |  |  |  |  |  |  |  |  |  |  |  |  |  |  |  |  |  |  |  |  |  |  |  |  |  | T05E11.2 |  |
|  |  |  |  |  |  |  |  |  |  |  |  |  |  |  |  |  |  |  |  |  |  |  |  |  |  |  |  |  |  |  |  |  |  |  |  |  |  | *lin-29* | abnormal cell LINeage |
|  |  |  |  |  |  |  |  |  |  |  |  |  |  |  |  |  |  |  |  |  |  |  |  |  |  |  |  |  |  |  |  |  |  |  |  |  |  | C18H9.1 |  |
|  |  |  |  |  |  |  |  |  |  |  |  |  |  |  |  |  |  |  |  |  |  |  |  |  |  |  |  |  |  |  |  |  |  |  |  |  |  | R13A5.15 |  |
|  |  |  |  |  |  |  |  |  |  |  |  |  |  |  |  |  |  |  |  |  |  |  |  |  |  |  |  |  |  |  |  |  |  |  |  |  |  | Y53F4B.20 |  |
|  |  |  |  |  |  |  |  |  |  |  |  |  |  |  |  |  |  |  |  |  |  |  |  |  |  |  |  |  |  |  |  |  |  |  |  |  |  | ZK792.4 |  |
|  |  |  |  |  |  |  |  |  |  |  |  |  |  |  |  |  |  |  |  |  |  |  |  |  |  |  |  |  |  |  |  |  |  |  |  |  |  | *mef-2* | related to vertebrate MEF2 transcription factor |
|  |  |  |  |  |  |  |  |  |  |  |  |  |  |  |  |  |  |  |  |  |  |  |  |  |  |  |  |  |  |  |  |  |  |  |  |  |  | *ace-4* | abnormal AcetylCholinEsterase |
|  |  |  |  |  |  |  |  |  |  |  |  |  |  |  |  |  |  |  |  |  |  |  |  |  |  |  |  |  |  |  |  |  |  |  |  |  |  | C17H1.1 |  |
|  |  |  |  |  |  |  |  |  |  |  |  |  |  |  |  |  |  |  |  |  |  |  |  |  |  |  |  |  |  |  |  |  |  |  |  |  |  | *nhr-125* | Nuclear Hormone Receptor family |
|  |  |  |  |  |  |  |  |  |  |  |  |  |  |  |  |  |  |  |  |  |  |  |  |  |  |  |  |  |  |  |  |  |  |  |  |  |  | Y39A3B.1 |  |
|  |  |  |  |  |  |  |  |  |  |  |  |  |  |  |  |  |  |  |  |  |  |  |  |  |  |  |  |  |  |  |  |  |  |  |  |  |  | F47F2.3 |  |
|  |  |  |  |  |  |  |  |  |  |  |  |  |  |  |  |  |  |  |  |  |  |  |  |  |  |  |  |  |  |  |  |  |  |  |  |  |  | *hum-10* | Heavy chain, Unconventional Myosin |
|  |  |  |  |  |  |  |  |  |  |  |  |  |  |  |  |  |  |  |  |  |  |  |  |  |  |  |  |  |  |  |  |  |  |  |  |  |  | C25F6.6 |  |
|  |  |  |  |  |  |  |  |  |  |  |  |  |  |  |  |  |  |  |  |  |  |  |  |  |  |  |  |  |  |  |  |  |  |  |  |  |  | R07E3.2 |  |
|  |  |  |  |  |  |  |  |  |  |  |  |  |  |  |  |  |  |  |  |  |  |  |  |  |  |  |  |  |  |  |  |  |  |  |  |  |  | B0348.5 |  |
|  |  |  |  |  |  |  |  |  |  |  |  |  |  |  |  |  |  |  |  |  |  |  |  |  |  |  |  |  |  |  |  |  |  |  |  |  |  | T22F7.5 |  |
|  |  |  |  |  |  |  |  |  |  |  |  |  |  |  |  |  |  |  |  |  |  |  |  |  |  |  |  |  |  |  |  |  |  |  |  |  |  | T09D3.3 |  |
|  |  |  |  |  |  |  |  |  |  |  |  |  |  |  |  |  |  |  |  |  |  |  |  |  |  |  |  |  |  |  |  |  |  |  |  |  |  | C30A5.10 |  |
|  |  |  |  |  |  |  |  |  |  |  |  |  |  |  |  |  |  |  |  |  |  |  |  |  |  |  |  |  |  |  |  |  |  |  |  |  |  | *pqn-67* | Prion-like-(Q/N-rich)-domain-bearing protein |
|  |  |  |  |  |  |  |  |  |  |  |  |  |  |  |  |  |  |  |  |  |  |  |  |  |  |  |  |  |  |  |  |  |  |  |  |  |  | *kin-14* | protein KINase |
|  |  |  |  |  |  |  |  |  |  |  |  |  |  |  |  |  |  |  |  |  |  |  |  |  |  |  |  |  |  |  |  |  |  |  |  |  |  | cTel3X.1 |  |
|  |  |  |  |  |  |  |  |  |  |  |  |  |  |  |  |  |  |  |  |  |  |  |  |  |  |  |  |  |  |  |  |  |  |  |  |  |  | F55H12.3 |  |
|  |  |  |  |  |  |  |  |  |  |  |  |  |  |  |  |  |  |  |  |  |  |  |  |  |  |  |  |  |  |  |  |  |  |  |  |  |  | *tnt-4* | TropoNin T |
|  |  |  |  |  |  |  |  |  |  |  |  |  |  |  |  |  |  |  |  |  |  |  |  |  |  |  |  |  |  |  |  |  |  |  |  |  |  | F23F12.8 |  |
|  |  |  |  |  |  |  |  |  |  |  |  |  |  |  |  |  |  |  |  |  |  |  |  |  |  |  |  |  |  |  |  |  |  |  |  |  |  | *twk-11* | TWiK family of potassium channels |
|  |  |  |  |  |  |  |  |  |  |  |  |  |  |  |  |  |  |  |  |  |  |  |  |  |  |  |  |  |  |  |  |  |  |  |  |  |  | D1054.9 |  |
|  |  |  |  |  |  |  |  |  |  |  |  |  |  |  |  |  |  |  |  |  |  |  |  |  |  |  |  |  |  |  |  |  |  |  |  |  |  | ZC455.1 |  |
|  |  |  |  |  |  |  |  |  |  |  |  |  |  |  |  |  |  |  |  |  |  |  |  |  |  |  |  |  |  |  |  |  |  |  |  |  |  | C34E11.2 |  |
|  |  |  |  |  |  |  |  |  |  |  |  |  |  |  |  |  |  |  |  |  |  |  |  |  |  |  |  |  |  |  |  |  |  |  |  |  |  | F41G3.2 |  |
|  |  |  |  |  |  |  |  |  |  |  |  |  |  |  |  |  |  |  |  |  |  |  |  |  |  |  |  |  |  |  |  |  |  |  |  |  |  | T05C12.4 |  |
|  |  |  |  |  |  |  |  |  |  |  |  |  |  |  |  |  |  |  |  |  |  |  |  |  |  |  |  |  |  |  |  |  |  |  |  |  |  | VB0395L.1 |  |
|  |  |  |  |  |  |  |  |  |  |  |  |  |  |  |  |  |  |  |  |  |  |  |  |  |  |  |  |  |  |  |  |  |  |  |  |  |  | F21E9.2 |  |
|  |  |  |  |  |  |  |  |  |  |  |  |  |  |  |  |  |  |  |  |  |  |  |  |  |  |  |  |  |  |  |  |  |  |  |  |  |  | ZK816.4 |  |
|  |  |  |  |  |  |  |  |  |  |  |  |  |  |  |  |  |  |  |  |  |  |  |  |  |  |  |  |  |  |  |  |  |  |  |  |  |  | C38C3.4 |  |
|  |  |  |  |  |  |  |  |  |  |  |  |  |  |  |  |  |  |  |  |  |  |  |  |  |  |  |  |  |  |  |  |  |  |  |  |  |  | C09B8.3 |  |
|  |  |  |  |  |  |  |  |  |  |  |  |  |  |  |  |  |  |  |  |  |  |  |  |  |  |  |  |  |  |  |  |  |  |  |  |  |  | F55G1.1 |  |
|  |  |  |  |  |  |  |  |  |  |  |  |  |  |  |  |  |  |  |  |  |  |  |  |  |  |  |  |  |  |  |  |  |  |  |  |  |  | F54D5.15 |  |
|  |  |  |  |  |  |  |  |  |  |  |  |  |  |  |  |  |  |  |  |  |  |  |  |  |  |  |  |  |  |  |  |  |  |  |  |  |  | *lrx-1* | LRP X(cross)-hybridizing |
|  |  |  |  |  |  |  |  |  |  |  |  |  |  |  |  |  |  |  |  |  |  |  |  |  |  |  |  |  |  |  |  |  |  |  |  |  |  | Y55F3BR.2 |  |
|  |  |  |  |  |  |  |  |  |  |  |  |  |  |  |  |  |  |  |  |  |  |  |  |  |  |  |  |  |  |  |  |  |  |  |  |  |  | C34F6.1 |  |
|  |  |  |  |  |  |  |  |  |  |  |  |  |  |  |  |  |  |  |  |  |  |  |  |  |  |  |  |  |  |  |  |  |  |  |  |  |  | ZK287.4 |  |
|  |  |  |  |  |  |  |  |  |  |  |  |  |  |  |  |  |  |  |  |  |  |  |  |  |  |  |  |  |  |  |  |  |  |  |  |  |  | C54G4.4 |  |
|  |  |  |  |  |  |  |  |  |  |  |  |  |  |  |  |  |  |  |  |  |  |  |  |  |  |  |  |  |  |  |  |  |  |  |  |  |  | *spp-7* | SaPosin-like Protein family |
|  |  |  |  |  |  |  |  |  |  |  |  |  |  |  |  |  |  |  |  |  |  |  |  |  |  |  |  |  |  |  |  |  |  |  |  |  |  | F52H2.6 |  |
|  |  |  |  |  |  |  |  |  |  |  |  |  |  |  |  |  |  |  |  |  |  |  |  |  |  |  |  |  |  |  |  |  |  |  |  |  |  | *akt-2* | AKT kinase family |
|  |  |  |  |  |  |  |  |  |  |  |  |  |  |  |  |  |  |  |  |  |  |  |  |  |  |  |  |  |  |  |  |  |  |  |  |  |  | *nhr-61* | Nuclear Hormone Receptor family |
|  |  |  |  |  |  |  |  |  |  |  |  |  |  |  |  |  |  |  |  |  |  |  |  |  |  |  |  |  |  |  |  |  |  |  |  |  |  | *mab-31* | Male ABnormal |
|  |  |  |  |  |  |  |  |  |  |  |  |  |  |  |  |  |  |  |  |  |  |  |  |  |  |  |  |  |  |  |  |  |  |  |  |  |  | *apm-1* | AdaPtin, Mu/medium chain (clathrin associated complex) |
|  |  |  |  |  |  |  |  |  |  |  |  |  |  |  |  |  |  |  |  |  |  |  |  |  |  |  |  |  |  |  |  |  |  |  |  |  |  | *sma-5* | SMAll |
|  |  |  |  |  |  |  |  |  |  |  |  |  |  |  |  |  |  |  |  |  |  |  |  |  |  |  |  |  |  |  |  |  |  |  |  |  |  | *inx-11* | INneXin |
|  |  |  |  |  |  |  |  |  |  |  |  |  |  |  |  |  |  |  |  |  |  |  |  |  |  |  |  |  |  |  |  |  |  |  |  |  |  | Y56A3A.7 |  |
|  |  |  |  |  |  |  |  |  |  |  |  |  |  |  |  |  |  |  |  |  |  |  |  |  |  |  |  |  |  |  |  |  |  |  |  |  |  | *plc-3* | PhosphoLipase C |
|  |  |  |  |  |  |  |  |  |  |  |  |  |  |  |  |  |  |  |  |  |  |  |  |  |  |  |  |  |  |  |  |  |  |  |  |  |  | *cyp-33A1* | CYtochrome P450 family |
|  |  |  |  |  |  |  |  |  |  |  |  |  |  |  |  |  |  |  |  |  |  |  |  |  |  |  |  |  |  |  |  |  |  |  |  |  |  | *spe-15* | defective SPErmatogenesis |
|  |  |  |  |  |  |  |  |  |  |  |  |  |  |  |  |  |  |  |  |  |  |  |  |  |  |  |  |  |  |  |  |  |  |  |  |  |  | H03A11.1 |  |
|  |  |  |  |  |  |  |  |  |  |  |  |  |  |  |  |  |  |  |  |  |  |  |  |  |  |  |  |  |  |  |  |  |  |  |  |  |  | F55D12.5 |  |
|  |  |  |  |  |  |  |  |  |  |  |  |  |  |  |  |  |  |  |  |  |  |  |  |  |  |  |  |  |  |  |  |  |  |  |  |  |  | F20G4.2 |  |
|  |  |  |  |  |  |  |  |  |  |  |  |  |  |  |  |  |  |  |  |  |  |  |  |  |  |  |  |  |  |  |  |  |  |  |  |  |  | *itr-1* | Inositol Triphosphate Receptor |
|  |  |  |  |  |  |  |  |  |  |  |  |  |  |  |  |  |  |  |  |  |  |  |  |  |  |  |  |  |  |  |  |  |  |  |  |  |  | *nhr-98* | Nuclear Hormone Receptor family |
|  |  |  |  |  |  |  |  |  |  |  |  |  |  |  |  |  |  |  |  |  |  |  |  |  |  |  |  |  |  |  |  |  |  |  |  |  |  | C44C10.9 |  |
|  |  |  |  |  |  |  |  |  |  |  |  |  |  |  |  |  |  |  |  |  |  |  |  |  |  |  |  |  |  |  |  |  |  |  |  |  |  | F53A9.9 |  |
|  |  |  |  |  |  |  |  |  |  |  |  |  |  |  |  |  |  |  |  |  |  |  |  |  |  |  |  |  |  |  |  |  |  |  |  |  |  | F25G6.7 |  |
|  |  |  |  |  |  |  |  |  |  |  |  |  |  |  |  |  |  |  |  |  |  |  |  |  |  |  |  |  |  |  |  |  |  |  |  |  |  | *nhr-28* | Nuclear Hormone Receptor family |
|  |  |  |  |  |  |  |  |  |  |  |  |  |  |  |  |  |  |  |  |  |  |  |  |  |  |  |  |  |  |  |  |  |  |  |  |  |  | *nhr-203* | Nuclear Hormone Receptor family |
|  |  |  |  |  |  |  |  |  |  |  |  |  |  |  |  |  |  |  |  |  |  |  |  |  |  |  |  |  |  |  |  |  |  |  |  |  |  | ZK742.4 |  |
|  |  |  |  |  |  |  |  |  |  |  |  |  |  |  |  |  |  |  |  |  |  |  |  |  |  |  |  |  |  |  |  |  |  |  |  |  |  | *nhr-62* | Nuclear Hormone Receptor family |
|  |  |  |  |  |  |  |  |  |  |  |  |  |  |  |  |  |  |  |  |  |  |  |  |  |  |  |  |  |  |  |  |  |  |  |  |  |  | Y48C3A.3 |  |
|  |  |  |  |  |  |  |  |  |  |  |  |  |  |  |  |  |  |  |  |  |  |  |  |  |  |  |  |  |  |  |  |  |  |  |  |  |  | F52F10.2 |  |
|  |  |  |  |  |  |  |  |  |  |  |  |  |  |  |  |  |  |  |  |  |  |  |  |  |  |  |  |  |  |  |  |  |  |  |  |  |  | C44C1.6 |  |
|  |  |  |  |  |  |  |  |  |  |  |  |  |  |  |  |  |  |  |  |  |  |  |  |  |  |  |  |  |  |  |  |  |  |  |  |  |  | F26E4.7 |  |
|  |  |  |  |  |  |  |  |  |  |  |  |  |  |  |  |  |  |  |  |  |  |  |  |  |  |  |  |  |  |  |  |  |  |  |  |  |  | *pha-4* | defective PHArynx development |
|  |  |  |  |  |  |  |  |  |  |  |  |  |  |  |  |  |  |  |  |  |  |  |  |  |  |  |  |  |  |  |  |  |  |  |  |  |  | T12G3.4 |  |
|  |  |  |  |  |  |  |  |  |  |  |  |  |  |  |  |  |  |  |  |  |  |  |  |  |  |  |  |  |  |  |  |  |  |  |  |  |  | K07C11.4 |  |
|  |  |  |  |  |  |  |  |  |  |  |  |  |  |  |  |  |  |  |  |  |  |  |  |  |  |  |  |  |  |  |  |  |  |  |  |  |  | ZK899.2 |  |
|  |  |  |  |  |  |  |  |  |  |  |  |  |  |  |  |  |  |  |  |  |  |  |  |  |  |  |  |  |  |  |  |  |  |  |  |  |  | C54E4.4 |  |
|  |  |  |  |  |  |  |  |  |  |  |  |  |  |  |  |  |  |  |  |  |  |  |  |  |  |  |  |  |  |  |  |  |  |  |  |  |  | ZK262.2 |  |
|  |  |  |  |  |  |  |  |  |  |  |  |  |  |  |  |  |  |  |  |  |  |  |  |  |  |  |  |  |  |  |  |  |  |  |  |  |  | F14B4.1 |  |
|  |  |  |  |  |  |  |  |  |  |  |  |  |  |  |  |  |  |  |  |  |  |  |  |  |  |  |  |  |  |  |  |  |  |  |  |  |  | T06D8.3 |  |
|  |  |  |  |  |  |  |  |  |  |  |  |  |  |  |  |  |  |  |  |  |  |  |  |  |  |  |  |  |  |  |  |  |  |  |  |  |  | *ugt-57* | UDP-GlucuronosylTransferase |
|  |  |  |  |  |  |  |  |  |  |  |  |  |  |  |  |  |  |  |  |  |  |  |  |  |  |  |  |  |  |  |  |  |  |  |  |  |  | C14B9.2 |  |
|  |  |  |  |  |  |  |  |  |  |  |  |  |  |  |  |  |  |  |  |  |  |  |  |  |  |  |  |  |  |  |  |  |  |  |  |  |  | *kin-33* | protein KINase |
|  |  |  |  |  |  |  |  |  |  |  |  |  |  |  |  |  |  |  |  |  |  |  |  |  |  |  |  |  |  |  |  |  |  |  |  |  |  | C53B7.3 |  |
|  |  |  |  |  |  |  |  |  |  |  |  |  |  |  |  |  |  |  |  |  |  |  |  |  |  |  |  |  |  |  |  |  |  |  |  |  |  | F28B4.4 |  |
|  |  |  |  |  |  |  |  |  |  |  |  |  |  |  |  |  |  |  |  |  |  |  |  |  |  |  |  |  |  |  |  |  |  |  |  |  |  | Y66D12A.24 |  |
|  |  |  |  |  |  |  |  |  |  |  |  |  |  |  |  |  |  |  |  |  |  |  |  |  |  |  |  |  |  |  |  |  |  |  |  |  |  | *pax-1* | PAX (Paired box) transcription factor |
|  |  |  |  |  |  |  |  |  |  |  |  |  |  |  |  |  |  |  |  |  |  |  |  |  |  |  |  |  |  |  |  |  |  |  |  |  |  | C45B11.6 |  |
|  |  |  |  |  |  |  |  |  |  |  |  |  |  |  |  |  |  |  |  |  |  |  |  |  |  |  |  |  |  |  |  |  |  |  |  |  |  | *par-3* | abnormal embryonic PARtitioning of cytoplasm |
|  |  |  |  |  |  |  |  |  |  |  |  |  |  |  |  |  |  |  |  |  |  |  |  |  |  |  |  |  |  |  |  |  |  |  |  |  |  | W02C12.1 |  |
|  |  |  |  |  |  |  |  |  |  |  |  |  |  |  |  |  |  |  |  |  |  |  |  |  |  |  |  |  |  |  |  |  |  |  |  |  |  | *eps-8* | EPS (human endocytosis) related |
|  |  |  |  |  |  |  |  |  |  |  |  |  |  |  |  |  |  |  |  |  |  |  |  |  |  |  |  |  |  |  |  |  |  |  |  |  |  | *ced-6* | CEll Death abnormality |
|  |  |  |  |  |  |  |  |  |  |  |  |  |  |  |  |  |  |  |  |  |  |  |  |  |  |  |  |  |  |  |  |  |  |  |  |  |  | *bli-4* | BLIstered cuticle |
|  |  |  |  |  |  |  |  |  |  |  |  |  |  |  |  |  |  |  |  |  |  |  |  |  |  |  |  |  |  |  |  |  |  |  |  |  |  | *chtl-1* | CHoline Transporter-Like |
|  |  |  |  |  |  |  |  |  |  |  |  |  |  |  |  |  |  |  |  |  |  |  |  |  |  |  |  |  |  |  |  |  |  |  |  |  |  | *ztf-16* | Zinc finger putative Transcription Factor family |
|  |  |  |  |  |  |  |  |  |  |  |  |  |  |  |  |  |  |  |  |  |  |  |  |  |  |  |  |  |  |  |  |  |  |  |  |  |  | *ajm-1* | Apical Junction Molecule |
|  |  |  |  |  |  |  |  |  |  |  |  |  |  |  |  |  |  |  |  |  |  |  |  |  |  |  |  |  |  |  |  |  |  |  |  |  |  | *peb-1* | Pharyngeal Enhancer Binding |
|  |  |  |  |  |  |  |  |  |  |  |  |  |  |  |  |  |  |  |  |  |  |  |  |  |  |  |  |  |  |  |  |  |  |  |  |  |  | *ifc-2* | Intermediate Filament, C |
|  |  |  |  |  |  |  |  |  |  |  |  |  |  |  |  |  |  |  |  |  |  |  |  |  |  |  |  |  |  |  |  |  |  |  |  |  |  | M88.4 |  |
|  |  |  |  |  |  |  |  |  |  |  |  |  |  |  |  |  |  |  |  |  |  |  |  |  |  |  |  |  |  |  |  |  |  |  |  |  |  | *asns-1* | ASparagiNe Synthetase |
|  |  |  |  |  |  |  |  |  |  |  |  |  |  |  |  |  |  |  |  |  |  |  |  |  |  |  |  |  |  |  |  |  |  |  |  |  |  | *frpr-4* | FMRFamide Peptide Receptor family |
|  |  |  |  |  |  |  |  |  |  |  |  |  |  |  |  |  |  |  |  |  |  |  |  |  |  |  |  |  |  |  |  |  |  |  |  |  |  | Y62F5A.10 |  |
|  |  |  |  |  |  |  |  |  |  |  |  |  |  |  |  |  |  |  |  |  |  |  |  |  |  |  |  |  |  |  |  |  |  |  |  |  |  | *sphk-1* | SPHingosine Kinase |
|  |  |  |  |  |  |  |  |  |  |  |  |  |  |  |  |  |  |  |  |  |  |  |  |  |  |  |  |  |  |  |  |  |  |  |  |  |  | T03F7.7 |  |
|  |  |  |  |  |  |  |  |  |  |  |  |  |  |  |  |  |  |  |  |  |  |  |  |  |  |  |  |  |  |  |  |  |  |  |  |  |  | T11A5.6 |  |
|  |  |  |  |  |  |  |  |  |  |  |  |  |  |  |  |  |  |  |  |  |  |  |  |  |  |  |  |  |  |  |  |  |  |  |  |  |  | *nhr-154* | Nuclear Hormone Receptor family |
|  |  |  |  |  |  |  |  |  |  |  |  |  |  |  |  |  |  |  |  |  |  |  |  |  |  |  |  |  |  |  |  |  |  |  |  |  |  | *nhr-153* | Nuclear Hormone Receptor family |
|  |  |  |  |  |  |  |  |  |  |  |  |  |  |  |  |  |  |  |  |  |  |  |  |  |  |  |  |  |  |  |  |  |  |  |  |  |  | F35G2.1 |  |
|  |  |  |  |  |  |  |  |  |  |  |  |  |  |  |  |  |  |  |  |  |  |  |  |  |  |  |  |  |  |  |  |  |  |  |  |  |  | T03D8.6 |  |
|  |  |  |  |  |  |  |  |  |  |  |  |  |  |  |  |  |  |  |  |  |  |  |  |  |  |  |  |  |  |  |  |  |  |  |  |  |  | *sem-2* | SEx Muscle abnormal |
|  |  |  |  |  |  |  |  |  |  |  |  |  |  |  |  |  |  |  |  |  |  |  |  |  |  |  |  |  |  |  |  |  |  |  |  |  |  | C09E8.1 |  |
|  |  |  |  |  |  |  |  |  |  |  |  |  |  |  |  |  |  |  |  |  |  |  |  |  |  |  |  |  |  |  |  |  |  |  |  |  |  | *unc-52* | UNCoordinated |
|  |  |  |  |  |  |  |  |  |  |  |  |  |  |  |  |  |  |  |  |  |  |  |  |  |  |  |  |  |  |  |  |  |  |  |  |  |  | *cpt-4* | Carnitine Palmitoyl Transferase |
|  |  |  |  |  |  |  |  |  |  |  |  |  |  |  |  |  |  |  |  |  |  |  |  |  |  |  |  |  |  |  |  |  |  |  |  |  |  | *lrp-1* | Low-density lipoprotein RecePtor related |
|  |  |  |  |  |  |  |  |  |  |  |  |  |  |  |  |  |  |  |  |  |  |  |  |  |  |  |  |  |  |  |  |  |  |  |  |  |  | *gei-13* | GEX Interacting protein |
|  |  |  |  |  |  |  |  |  |  |  |  |  |  |  |  |  |  |  |  |  |  |  |  |  |  |  |  |  |  |  |  |  |  |  |  |  |  | C10G8.8 |  |
|  |  |  |  |  |  |  |  |  |  |  |  |  |  |  |  |  |  |  |  |  |  |  |  |  |  |  |  |  |  |  |  |  |  |  |  |  |  | *hum-6* | Heavy chain, Unconventional Myosin |
|  |  |  |  |  |  |  |  |  |  |  |  |  |  |  |  |  |  |  |  |  |  |  |  |  |  |  |  |  |  |  |  |  |  |  |  |  |  | *obr-1* | Oxysterol Binding protein (OSBP) Related |
|  |  |  |  |  |  |  |  |  |  |  |  |  |  |  |  |  |  |  |  |  |  |  |  |  |  |  |  |  |  |  |  |  |  |  |  |  |  | *ets-4* | ETS class transcription factor |
|  |  |  |  |  |  |  |  |  |  |  |  |  |  |  |  |  |  |  |  |  |  |  |  |  |  |  |  |  |  |  |  |  |  |  |  |  |  | *yap-1* | Yes-Associated Protein homolog |
|  |  |  |  |  |  |  |  |  |  |  |  |  |  |  |  |  |  |  |  |  |  |  |  |  |  |  |  |  |  |  |  |  |  |  |  |  |  | *dpy-31* | DumPY: shorter than wild-type |
|  |  |  |  |  |  |  |  |  |  |  |  |  |  |  |  |  |  |  |  |  |  |  |  |  |  |  |  |  |  |  |  |  |  |  |  |  |  | T24B8.4 |  |
|  |  |  |  |  |  |  |  |  |  |  |  |  |  |  |  |  |  |  |  |  |  |  |  |  |  |  |  |  |  |  |  |  |  |  |  |  |  | *dhs-16* | DeHydrogenases, Short chain |
|  |  |  |  |  |  |  |  |  |  |  |  |  |  |  |  |  |  |  |  |  |  |  |  |  |  |  |  |  |  |  |  |  |  |  |  |  |  | *nex-1* | anNEXin family |
|  |  |  |  |  |  |  |  |  |  |  |  |  |  |  |  |  |  |  |  |  |  |  |  |  |  |  |  |  |  |  |  |  |  |  |  |  |  | *nhr-202* | Nuclear Hormone Receptor family |
|  |  |  |  |  |  |  |  |  |  |  |  |  |  |  |  |  |  |  |  |  |  |  |  |  |  |  |  |  |  |  |  |  |  |  |  |  |  | *nhr-201* | Nuclear Hormone Receptor family |
|  |  |  |  |  |  |  |  |  |  |  |  |  |  |  |  |  |  |  |  |  |  |  |  |  |  |  |  |  |  |  |  |  |  |  |  |  |  | H16D19.4 |  |
|  |  |  |  |  |  |  |  |  |  |  |  |  |  |  |  |  |  |  |  |  |  |  |  |  |  |  |  |  |  |  |  |  |  |  |  |  |  | *str-243* | Seven TM Receptor |
|  |  |  |  |  |  |  |  |  |  |  |  |  |  |  |  |  |  |  |  |  |  |  |  |  |  |  |  |  |  |  |  |  |  |  |  |  |  | *ethe-1* | ETHE1 (human ETHylmalonic Encephalopathy) homolog |
|  |  |  |  |  |  |  |  |  |  |  |  |  |  |  |  |  |  |  |  |  |  |  |  |  |  |  |  |  |  |  |  |  |  |  |  |  |  | K10C2.2 |  |
|  |  |  |  |  |  |  |  |  |  |  |  |  |  |  |  |  |  |  |  |  |  |  |  |  |  |  |  |  |  |  |  |  |  |  |  |  |  | *klp-6* | Kinesin-Like Protein |
|  |  |  |  |  |  |  |  |  |  |  |  |  |  |  |  |  |  |  |  |  |  |  |  |  |  |  |  |  |  |  |  |  |  |  |  |  |  | *eak-4* | Enhancer of AKt-1 null |
|  |  |  |  |  |  |  |  |  |  |  |  |  |  |  |  |  |  |  |  |  |  |  |  |  |  |  |  |  |  |  |  |  |  |  |  |  |  | *grl-17* | GRound-Like (grd related) |
|  |  |  |  |  |  |  |  |  |  |  |  |  |  |  |  |  |  |  |  |  |  |  |  |  |  |  |  |  |  |  |  |  |  |  |  |  |  | *lgc-10* | Ligand-Gated ion Channel |
|  |  |  |  |  |  |  |  |  |  |  |  |  |  |  |  |  |  |  |  |  |  |  |  |  |  |  |  |  |  |  |  |  |  |  |  |  |  | *arrd-23* | ARRestin Domain protein |
|  |  |  |  |  |  |  |  |  |  |  |  |  |  |  |  |  |  |  |  |  |  |  |  |  |  |  |  |  |  |  |  |  |  |  |  |  |  | B0218.5 |  |
|  |  |  |  |  |  |  |  |  |  |  |  |  |  |  |  |  |  |  |  |  |  |  |  |  |  |  |  |  |  |  |  |  |  |  |  |  |  | *clic-1* | Clathrin LIght Chain |
|  |  |  |  |  |  |  |  |  |  |  |  |  |  |  |  |  |  |  |  |  |  |  |  |  |  |  |  |  |  |  |  |  |  |  |  |  |  | *daam-1* | DAAM (Disheveled-Associated Activator of Morphogenesis) homolog |
|  |  |  |  |  |  |  |  |  |  |  |  |  |  |  |  |  |  |  |  |  |  |  |  |  |  |  |  |  |  |  |  |  |  |  |  |  |  | K10D6.4 |  |
|  |  |  |  |  |  |  |  |  |  |  |  |  |  |  |  |  |  |  |  |  |  |  |  |  |  |  |  |  |  |  |  |  |  |  |  |  |  | C33A11.1 |  |
|  |  |  |  |  |  |  |  |  |  |  |  |  |  |  |  |  |  |  |  |  |  |  |  |  |  |  |  |  |  |  |  |  |  |  |  |  |  | *sre-34* | Serpentine Receptor, class E (epsilon) |
|  |  |  |  |  |  |  |  |  |  |  |  |  |  |  |  |  |  |  |  |  |  |  |  |  |  |  |  |  |  |  |  |  |  |  |  |  |  | *ttx-1* | abnormal ThermoTaXis |
|  |  |  |  |  |  |  |  |  |  |  |  |  |  |  |  |  |  |  |  |  |  |  |  |  |  |  |  |  |  |  |  |  |  |  |  |  |  | F09C6.13 |  |
|  |  |  |  |  |  |  |  |  |  |  |  |  |  |  |  |  |  |  |  |  |  |  |  |  |  |  |  |  |  |  |  |  |  |  |  |  |  | ZK816.3 |  |
|  |  |  |  |  |  |  |  |  |  |  |  |  |  |  |  |  |  |  |  |  |  |  |  |  |  |  |  |  |  |  |  |  |  |  |  |  |  | ZK402.3 |  |
|  |  |  |  |  |  |  |  |  |  |  |  |  |  |  |  |  |  |  |  |  |  |  |  |  |  |  |  |  |  |  |  |  |  |  |  |  |  | C55F2.1 |  |
|  |  |  |  |  |  |  |  |  |  |  |  |  |  |  |  |  |  |  |  |  |  |  |  |  |  |  |  |  |  |  |  |  |  |  |  |  |  | F09C6.12 |  |
|  |  |  |  |  |  |  |  |  |  |  |  |  |  |  |  |  |  |  |  |  |  |  |  |  |  |  |  |  |  |  |  |  |  |  |  |  |  | *sre-30* | Serpentine Receptor, class E (epsilon) |
|  |  |  |  |  |  |  |  |  |  |  |  |  |  |  |  |  |  |  |  |  |  |  |  |  |  |  |  |  |  |  |  |  |  |  |  |  |  | *lipl-4* | LIPase Like |
|  |  |  |  |  |  |  |  |  |  |  |  |  |  |  |  |  |  |  |  |  |  |  |  |  |  |  |  |  |  |  |  |  |  |  |  |  |  | *fbxa-100* | F-box A protein |
|  |  |  |  |  |  |  |  |  |  |  |  |  |  |  |  |  |  |  |  |  |  |  |  |  |  |  |  |  |  |  |  |  |  |  |  |  |  | *cdh-10* | CaDHerin family |
|  |  |  |  |  |  |  |  |  |  |  |  |  |  |  |  |  |  |  |  |  |  |  |  |  |  |  |  |  |  |  |  |  |  |  |  |  |  | F13C5.3 |  |
|  |  |  |  |  |  |  |  |  |  |  |  |  |  |  |  |  |  |  |  |  |  |  |  |  |  |  |  |  |  |  |  |  |  |  |  |  |  | F38A1.13 |  |
|  |  |  |  |  |  |  |  |  |  |  |  |  |  |  |  |  |  |  |  |  |  |  |  |  |  |  |  |  |  |  |  |  |  |  |  |  |  | T09B4.3 |  |
|  |  |  |  |  |  |  |  |  |  |  |  |  |  |  |  |  |  |  |  |  |  |  |  |  |  |  |  |  |  |  |  |  |  |  |  |  |  | Y37H2C.1 |  |
|  |  |  |  |  |  |  |  |  |  |  |  |  |  |  |  |  |  |  |  |  |  |  |  |  |  |  |  |  |  |  |  |  |  |  |  |  |  | C44B11.4 |  |
|  |  |  |  |  |  |  |  |  |  |  |  |  |  |  |  |  |  |  |  |  |  |  |  |  |  |  |  |  |  |  |  |  |  |  |  |  |  | T01B11.1 |  |
|  |  |  |  |  |  |  |  |  |  |  |  |  |  |  |  |  |  |  |  |  |  |  |  |  |  |  |  |  |  |  |  |  |  |  |  |  |  | *gst-31* | Glutathione S-Transferase |
|  |  |  |  |  |  |  |  |  |  |  |  |  |  |  |  |  |  |  |  |  |  |  |  |  |  |  |  |  |  |  |  |  |  |  |  |  |  | *srxa-15* | Serpentine Receptor, class XA |
|  |  |  |  |  |  |  |  |  |  |  |  |  |  |  |  |  |  |  |  |  |  |  |  |  |  |  |  |  |  |  |  |  |  |  |  |  |  | *asic-2* | Acid-sensing/Amiloride-Sensitive Ion Channel family |
|  |  |  |  |  |  |  |  |  |  |  |  |  |  |  |  |  |  |  |  |  |  |  |  |  |  |  |  |  |  |  |  |  |  |  |  |  |  | *srab-24* | Serpentine Receptor, class AB (class A-like) |
|  |  |  |  |  |  |  |  |  |  |  |  |  |  |  |  |  |  |  |  |  |  |  |  |  |  |  |  |  |  |  |  |  |  |  |  |  |  | *srbc-33* | Serpentine Receptor, class BC (class B-like) |
|  |  |  |  |  |  |  |  |  |  |  |  |  |  |  |  |  |  |  |  |  |  |  |  |  |  |  |  |  |  |  |  |  |  |  |  |  |  | *sre-6* | Serpentine Receptor, class E (epsilon) |
|  |  |  |  |  |  |  |  |  |  |  |  |  |  |  |  |  |  |  |  |  |  |  |  |  |  |  |  |  |  |  |  |  |  |  |  |  |  | *gpa-6* | G Protein, Alpha subunit |
|  |  |  |  |  |  |  |  |  |  |  |  |  |  |  |  |  |  |  |  |  |  |  |  |  |  |  |  |  |  |  |  |  |  |  |  |  |  | *irld-11* | Insulin/EGF-Receptor L Domain protein |
|  |  |  |  |  |  |  |  |  |  |  |  |  |  |  |  |  |  |  |  |  |  |  |  |  |  |  |  |  |  |  |  |  |  |  |  |  |  | *lurp-4* | LU (Ly6 Urokinase plasminogen) domain Receptor-related Protein |
|  |  |  |  |  |  |  |  |  |  |  |  |  |  |  |  |  |  |  |  |  |  |  |  |  |  |  |  |  |  |  |  |  |  |  |  |  |  | *cyp-25A3* | CYtochrome P450 family |
|  |  |  |  |  |  |  |  |  |  |  |  |  |  |  |  |  |  |  |  |  |  |  |  |  |  |  |  |  |  |  |  |  |  |  |  |  |  | *fbxa-69* | F-box A protein |
|  |  |  |  |  |  |  |  |  |  |  |  |  |  |  |  |  |  |  |  |  |  |  |  |  |  |  |  |  |  |  |  |  |  |  |  |  |  | *pqn-29* | Prion-like-(Q/N-rich)-domain-bearing protein |
|  |  |  |  |  |  |  |  |  |  |  |  |  |  |  |  |  |  |  |  |  |  |  |  |  |  |  |  |  |  |  |  |  |  |  |  |  |  | Y73B6A.1 |  |
|  |  |  |  |  |  |  |  |  |  |  |  |  |  |  |  |  |  |  |  |  |  |  |  |  |  |  |  |  |  |  |  |  |  |  |  |  |  | C44B12.6 |  |
|  |  |  |  |  |  |  |  |  |  |  |  |  |  |  |  |  |  |  |  |  |  |  |  |  |  |  |  |  |  |  |  |  |  |  |  |  |  | *acr-10* | AcetylCholine Receptor |
|  |  |  |  |  |  |  |  |  |  |  |  |  |  |  |  |  |  |  |  |  |  |  |  |  |  |  |  |  |  |  |  |  |  |  |  |  |  | *nas-8* | Nematode AStacin protease |
|  |  |  |  |  |  |  |  |  |  |  |  |  |  |  |  |  |  |  |  |  |  |  |  |  |  |  |  |  |  |  |  |  |  |  |  |  |  | T06E4.10 |  |
|  |  |  |  |  |  |  |  |  |  |  |  |  |  |  |  |  |  |  |  |  |  |  |  |  |  |  |  |  |  |  |  |  |  |  |  |  |  | T06E4.14 |  |
|  |  |  |  |  |  |  |  |  |  |  |  |  |  |  |  |  |  |  |  |  |  |  |  |  |  |  |  |  |  |  |  |  |  |  |  |  |  | C49F8.1 |  |
|  |  |  |  |  |  |  |  |  |  |  |  |  |  |  |  |  |  |  |  |  |  |  |  |  |  |  |  |  |  |  |  |  |  |  |  |  |  | Y65B4A.2 |  |
|  |  |  |  |  |  |  |  |  |  |  |  |  |  |  |  |  |  |  |  |  |  |  |  |  |  |  |  |  |  |  |  |  |  |  |  |  |  | C26B2.8 |  |
|  |  |  |  |  |  |  |  |  |  |  |  |  |  |  |  |  |  |  |  |  |  |  |  |  |  |  |  |  |  |  |  |  |  |  |  |  |  | Y61A9LA.7 |  |
|  |  |  |  |  |  |  |  |  |  |  |  |  |  |  |  |  |  |  |  |  |  |  |  |  |  |  |  |  |  |  |  |  |  |  |  |  |  | ZK470.6 |  |
|  |  |  |  |  |  |  |  |  |  |  |  |  |  |  |  |  |  |  |  |  |  |  |  |  |  |  |  |  |  |  |  |  |  |  |  |  |  | F16H11.1 |  |
|  |  |  |  |  |  |  |  |  |  |  |  |  |  |  |  |  |  |  |  |  |  |  |  |  |  |  |  |  |  |  |  |  |  |  |  |  |  | C35B8.4 |  |
|  |  |  |  |  |  |  |  |  |  |  |  |  |  |  |  |  |  |  |  |  |  |  |  |  |  |  |  |  |  |  |  |  |  |  |  |  |  | ZC328.2 |  |
|  |  |  |  |  |  |  |  |  |  |  |  |  |  |  |  |  |  |  |  |  |  |  |  |  |  |  |  |  |  |  |  |  |  |  |  |  |  | *nas-13* | Nematode AStacin protease |
|  |  |  |  |  |  |  |  |  |  |  |  |  |  |  |  |  |  |  |  |  |  |  |  |  |  |  |  |  |  |  |  |  |  |  |  |  |  | D1007.13 |  |
|  |  |  |  |  |  |  |  |  |  |  |  |  |  |  |  |  |  |  |  |  |  |  |  |  |  |  |  |  |  |  |  |  |  |  |  |  |  | ZC449.5 |  |
|  |  |  |  |  |  |  |  |  |  |  |  |  |  |  |  |  |  |  |  |  |  |  |  |  |  |  |  |  |  |  |  |  |  |  |  |  |  | *pqn-35* | Prion-like-(Q/N-rich)-domain-bearing protein |
|  |  |  |  |  |  |  |  |  |  |  |  |  |  |  |  |  |  |  |  |  |  |  |  |  |  |  |  |  |  |  |  |  |  |  |  |  |  | *gcy-22* | Guanylyl CYclase |
|  |  |  |  |  |  |  |  |  |  |  |  |  |  |  |  |  |  |  |  |  |  |  |  |  |  |  |  |  |  |  |  |  |  |  |  |  |  | T06E4.9 |  |
|  |  |  |  |  |  |  |  |  |  |  |  |  |  |  |  |  |  |  |  |  |  |  |  |  |  |  |  |  |  |  |  |  |  |  |  |  |  | *lron-6* | eLRR (extracellular Leucine-Rich Repeat) ONly |
|  |  |  |  |  |  |  |  |  |  |  |  |  |  |  |  |  |  |  |  |  |  |  |  |  |  |  |  |  |  |  |  |  |  |  |  |  |  | *nmy-1* | Non-muscle MYosin |
|  |  |  |  |  |  |  |  |  |  |  |  |  |  |  |  |  |  |  |  |  |  |  |  |  |  |  |  |  |  |  |  |  |  |  |  |  |  | F10G8.8 |  |
|  |  |  |  |  |  |  |  |  |  |  |  |  |  |  |  |  |  |  |  |  |  |  |  |  |  |  |  |  |  |  |  |  |  |  |  |  |  | F46C5.1 |  |
|  |  |  |  |  |  |  |  |  |  |  |  |  |  |  |  |  |  |  |  |  |  |  |  |  |  |  |  |  |  |  |  |  |  |  |  |  |  | *haf-7* | HAlF transporter (PGP related) |
|  |  |  |  |  |  |  |  |  |  |  |  |  |  |  |  |  |  |  |  |  |  |  |  |  |  |  |  |  |  |  |  |  |  |  |  |  |  | *tag-256* | Temporarily Assigned Gene name |
|  |  |  |  |  |  |  |  |  |  |  |  |  |  |  |  |  |  |  |  |  |  |  |  |  |  |  |  |  |  |  |  |  |  |  |  |  |  | *rme-1* | Receptor Mediated Endocytosis |
|  |  |  |  |  |  |  |  |  |  |  |  |  |  |  |  |  |  |  |  |  |  |  |  |  |  |  |  |  |  |  |  |  |  |  |  |  |  | *exp-2* | EXPulsion defective (defecation) |
|  |  |  |  |  |  |  |  |  |  |  |  |  |  |  |  |  |  |  |  |  |  |  |  |  |  |  |  |  |  |  |  |  |  |  |  |  |  | *tmc-1* | TMC (TransMembrane Channel-like protein) family |
|  |  |  |  |  |  |  |  |  |  |  |  |  |  |  |  |  |  |  |  |  |  |  |  |  |  |  |  |  |  |  |  |  |  |  |  |  |  | *pac-1* | PAR-6 At Contacts (abnormal early localization of PAR-6) |
|  |  |  |  |  |  |  |  |  |  |  |  |  |  |  |  |  |  |  |  |  |  |  |  |  |  |  |  |  |  |  |  |  |  |  |  |  |  | *rgs-2* | Regulator of G protein Signaling |
|  |  |  |  |  |  |  |  |  |  |  |  |  |  |  |  |  |  |  |  |  |  |  |  |  |  |  |  |  |  |  |  |  |  |  |  |  |  | *kin-29* | protein KINase |
|  |  |  |  |  |  |  |  |  |  |  |  |  |  |  |  |  |  |  |  |  |  |  |  |  |  |  |  |  |  |  |  |  |  |  |  |  |  | Y67D8C.9 |  |
|  |  |  |  |  |  |  |  |  |  |  |  |  |  |  |  |  |  |  |  |  |  |  |  |  |  |  |  |  |  |  |  |  |  |  |  |  |  | *tbc-17* | TBC (Tre-2/Bub2/Cdc16) domain family |
|  |  |  |  |  |  |  |  |  |  |  |  |  |  |  |  |  |  |  |  |  |  |  |  |  |  |  |  |  |  |  |  |  |  |  |  |  |  | *scd-1* | Suppressor of Constitutive Dauer formation |
|  |  |  |  |  |  |  |  |  |  |  |  |  |  |  |  |  |  |  |  |  |  |  |  |  |  |  |  |  |  |  |  |  |  |  |  |  |  | *apl-1* | Amyloid Precursor-Like |
|  |  |  |  |  |  |  |  |  |  |  |  |  |  |  |  |  |  |  |  |  |  |  |  |  |  |  |  |  |  |  |  |  |  |  |  |  |  | *nhr-71* | Nuclear Hormone Receptor family |
|  |  |  |  |  |  |  |  |  |  |  |  |  |  |  |  |  |  |  |  |  |  |  |  |  |  |  |  |  |  |  |  |  |  |  |  |  |  | *shn-1* | SHaNk (SH3/ankyrin domain scaffold protein) related |
|  |  |  |  |  |  |  |  |  |  |  |  |  |  |  |  |  |  |  |  |  |  |  |  |  |  |  |  |  |  |  |  |  |  |  |  |  |  | *gap-2* | GTPase Activating Protein family |
|  |  |  |  |  |  |  |  |  |  |  |  |  |  |  |  |  |  |  |  |  |  |  |  |  |  |  |  |  |  |  |  |  |  |  |  |  |  | *ser-4* | SERotonin/octopamine receptor family |
|  |  |  |  |  |  |  |  |  |  |  |  |  |  |  |  |  |  |  |  |  |  |  |  |  |  |  |  |  |  |  |  |  |  |  |  |  |  | *nhr-66* | Nuclear Hormone Receptor family |
|  |  |  |  |  |  |  |  |  |  |  |  |  |  |  |  |  |  |  |  |  |  |  |  |  |  |  |  |  |  |  |  |  |  |  |  |  |  | Y37F4.5 |  |
|  |  |  |  |  |  |  |  |  |  |  |  |  |  |  |  |  |  |  |  |  |  |  |  |  |  |  |  |  |  |  |  |  |  |  |  |  |  | *dapk-1* | DAP (Death-Associated Protein) Kinase homolog |
|  |  |  |  |  |  |  |  |  |  |  |  |  |  |  |  |  |  |  |  |  |  |  |  |  |  |  |  |  |  |  |  |  |  |  |  |  |  | C34F6.10 |  |
|  |  |  |  |  |  |  |  |  |  |  |  |  |  |  |  |  |  |  |  |  |  |  |  |  |  |  |  |  |  |  |  |  |  |  |  |  |  | *nhr-49* | Nuclear Hormone Receptor family |
|  |  |  |  |  |  |  |  |  |  |  |  |  |  |  |  |  |  |  |  |  |  |  |  |  |  |  |  |  |  |  |  |  |  |  |  |  |  | F55C12.1 |  |
|  |  |  |  |  |  |  |  |  |  |  |  |  |  |  |  |  |  |  |  |  |  |  |  |  |  |  |  |  |  |  |  |  |  |  |  |  |  | *glr-7* | GLutamate Receptor family (AMPA) |
|  |  |  |  |  |  |  |  |  |  |  |  |  |  |  |  |  |  |  |  |  |  |  |  |  |  |  |  |  |  |  |  |  |  |  |  |  |  | C30H7.2 |  |
|  |  |  |  |  |  |  |  |  |  |  |  |  |  |  |  |  |  |  |  |  |  |  |  |  |  |  |  |  |  |  |  |  |  |  |  |  |  | *cyld-1* | CYLinDromatosis (human disease gene) homolog |
|  |  |  |  |  |  |  |  |  |  |  |  |  |  |  |  |  |  |  |  |  |  |  |  |  |  |  |  |  |  |  |  |  |  |  |  |  |  | ZC15.4 |  |
|  |  |  |  |  |  |  |  |  |  |  |  |  |  |  |  |  |  |  |  |  |  |  |  |  |  |  |  |  |  |  |  |  |  |  |  |  |  | *cal-8* | CALmodulin related genes |
|  |  |  |  |  |  |  |  |  |  |  |  |  |  |  |  |  |  |  |  |  |  |  |  |  |  |  |  |  |  |  |  |  |  |  |  |  |  | ZC15.5 |  |
|  |  |  |  |  |  |  |  |  |  |  |  |  |  |  |  |  |  |  |  |  |  |  |  |  |  |  |  |  |  |  |  |  |  |  |  |  |  | *nhr-41* | Nuclear Hormone Receptor family |
|  |  |  |  |  |  |  |  |  |  |  |  |  |  |  |  |  |  |  |  |  |  |  |  |  |  |  |  |  |  |  |  |  |  |  |  |  |  | Y43E12A.2 |  |
|  |  |  |  |  |  |  |  |  |  |  |  |  |  |  |  |  |  |  |  |  |  |  |  |  |  |  |  |  |  |  |  |  |  |  |  |  |  | *srf-3* | SuRFace antigenicity abnormal |
|  |  |  |  |  |  |  |  |  |  |  |  |  |  |  |  |  |  |  |  |  |  |  |  |  |  |  |  |  |  |  |  |  |  |  |  |  |  | F11D5.1 |  |
|  |  |  |  |  |  |  |  |  |  |  |  |  |  |  |  |  |  |  |  |  |  |  |  |  |  |  |  |  |  |  |  |  |  |  |  |  |  | *fos-1* | FOS (B-Zip transcription factor) homolog |
|  |  |  |  |  |  |  |  |  |  |  |  |  |  |  |  |  |  |  |  |  |  |  |  |  |  |  |  |  |  |  |  |  |  |  |  |  |  | *nhr-58* | Nuclear Hormone Receptor family |
|  |  |  |  |  |  |  |  |  |  |  |  |  |  |  |  |  |  |  |  |  |  |  |  |  |  |  |  |  |  |  |  |  |  |  |  |  |  | C45G9.13 |  |
|  |  |  |  |  |  |  |  |  |  |  |  |  |  |  |  |  |  |  |  |  |  |  |  |  |  |  |  |  |  |  |  |  |  |  |  |  |  | ZK1128.7 |  |
|  |  |  |  |  |  |  |  |  |  |  |  |  |  |  |  |  |  |  |  |  |  |  |  |  |  |  |  |  |  |  |  |  |  |  |  |  |  | D1053.4 |  |
|  |  |  |  |  |  |  |  |  |  |  |  |  |  |  |  |  |  |  |  |  |  |  |  |  |  |  |  |  |  |  |  |  |  |  |  |  |  | *fbxa-34* | F-box A protein |
|  |  |  |  |  |  |  |  |  |  |  |  |  |  |  |  |  |  |  |  |  |  |  |  |  |  |  |  |  |  |  |  |  |  |  |  |  |  | F57B1.5 |  |
|  |  |  |  |  |  |  |  |  |  |  |  |  |  |  |  |  |  |  |  |  |  |  |  |  |  |  |  |  |  |  |  |  |  |  |  |  |  | *mans-2* | MAnnoSidase (family 47 glycohydrolase) |
|  |  |  |  |  |  |  |  |  |  |  |  |  |  |  |  |  |  |  |  |  |  |  |  |  |  |  |  |  |  |  |  |  |  |  |  |  |  | *inx-10* | INneXin |
|  |  |  |  |  |  |  |  |  |  |  |  |  |  |  |  |  |  |  |  |  |  |  |  |  |  |  |  |  |  |  |  |  |  |  |  |  |  | Y34B4A.4 |  |
|  |  |  |  |  |  |  |  |  |  |  |  |  |  |  |  |  |  |  |  |  |  |  |  |  |  |  |  |  |  |  |  |  |  |  |  |  |  | F55D12.2 |  |
|  |  |  |  |  |  |  |  |  |  |  |  |  |  |  |  |  |  |  |  |  |  |  |  |  |  |  |  |  |  |  |  |  |  |  |  |  |  | F25A2.1 |  |
|  |  |  |  |  |  |  |  |  |  |  |  |  |  |  |  |  |  |  |  |  |  |  |  |  |  |  |  |  |  |  |  |  |  |  |  |  |  | ZK1290.14 |  |
|  |  |  |  |  |  |  |  |  |  |  |  |  |  |  |  |  |  |  |  |  |  |  |  |  |  |  |  |  |  |  |  |  |  |  |  |  |  | Y55F3AM.10 |  |
|  |  |  |  |  |  |  |  |  |  |  |  |  |  |  |  |  |  |  |  |  |  |  |  |  |  |  |  |  |  |  |  |  |  |  |  |  |  | F46H6.5 |  |
|  |  |  |  |  |  |  |  |  |  |  |  |  |  |  |  |  |  |  |  |  |  |  |  |  |  |  |  |  |  |  |  |  |  |  |  |  |  | *dmd-6* | DM (Doublesex/MAB-3) Domain family |
|  |  |  |  |  |  |  |  |  |  |  |  |  |  |  |  |  |  |  |  |  |  |  |  |  |  |  |  |  |  |  |  |  |  |  |  |  |  | *nhr-3* | Nuclear Hormone Receptor family |
|  |  |  |  |  |  |  |  |  |  |  |  |  |  |  |  |  |  |  |  |  |  |  |  |  |  |  |  |  |  |  |  |  |  |  |  |  |  | *zip-2* | bZIP transcription factor family |
|  |  |  |  |  |  |  |  |  |  |  |  |  |  |  |  |  |  |  |  |  |  |  |  |  |  |  |  |  |  |  |  |  |  |  |  |  |  | *nhr-116* | Nuclear Hormone Receptor family |

### Phenotypes enriched

none found

### Anatomy terms enriched

|  |  |  |  |
| --- | --- | --- | --- |
| **Group name** | **Number in cluster** | **Enrichment** | **FDR corrected p** |
| pharynx | 52 | 1.90 | 0.00905 |
| epithelial cell | 33 | 2.30 | 0.01440 |
| rectal epithelium | 14 | 3.93 | 0.02590 |
| pharyngeal cell | 25 | 2.48 | 0.04370 |

### GO terms enriched

|  |  |  |
| --- | --- | --- |
| **GO term** | **Number of genes** | **FDR-corrected p-value** |
| cellular response to hormone stimulus | 20 | 9.4e-06 |
| response to endogenous stimulus | 20 | 1.2e-05 |
| sequence-specific DNA binding | 27 | 1.7e-05 |
| response to organic substance | 22 | 1.7e-05 |
| steroid hormone mediated signaling pathway | 19 | 1.8e-05 |
| response to steroid hormone | 19 | 1.8e-05 |
| cellular response to lipid | 19 | 1.8e-05 |
| cellular response to chemical stimulus | 22 | 2.1e-05 |
| cellular response to organic cyclic compound | 19 | 2.8e-05 |
| steroid hormone receptor activity | 19 | 5.5e-05 |
| signaling | 38 | 5.3e-04 |
| signal transduction | 36 | 5.4e-04 |
| regulation of nitrogen compound metabolic process | 31 | 7.7e-04 |
| sequence-specific DNA binding transcription factor activity | 24 | 1.4e-03 |
| regulation of transcription, DNA-templated | 28 | 1.7e-03 |
| regulation of RNA metabolic process | 28 | 1.9e-03 |
| regulation of cellular biosynthetic process | 29 | 2.3e-03 |
| regulation of primary metabolic process | 32 | 2.7e-03 |
| cellular nitrogen compound biosynthetic process | 31 | 3.7e-03 |
| signal transducer activity | 27 | 4.2e-03 |
| regulation of macromolecule biosynthetic process | 28 | 5.1e-03 |
| aromatic compound biosynthetic process | 30 | 6.5e-03 |
| receptor activity | 25 | 6.7e-03 |
| heterocycle biosynthetic process | 30 | 7.5e-03 |
| organic cyclic compound biosynthetic process | 30 | 9.7e-03 |
| RNA biosynthetic process | 24 | 1.2e-02 |
| zinc ion binding | 22 | 4.3e-02 |

### Expression clusters enriched

|  |  |  |  |
| --- | --- | --- | --- |
| **Group name** | **Number in cluster** | **Enrichment** | **FDR corrected p** |
| Genes up regulated by mir-243(n4759). | 73 | 2.31 | 2.44e-09 |
| Total muscle depleted genes (complete list of non-overlapping genes from the 0hr and 24hr muscle depleted datasets). | 89 | 2.04 | 4.65e-09 |
| Genes depleted in muscle cells (0hr muscle dataset). Dissociated myo-3::GFP embryos were cultured for 0 hours before FACS sorting. | 67 | 2.24 | 9.26e-08 |
| Genes differentially expressed in control vs after UVC exposure and EtBr treatment at the -1h timepoint (just prior to the third UVC dose (48h)). | 137 | 1.57 | 1.97e-07 |
| Genes differentially expressed under EtBr treatment and UVC exposure vs under UVC exposure but without EtBr treatment at the -1h timepoint (just prior to the third UVC dose (48h)). | 117 | 1.67 | 2.13e-07 |
| Genes upregulated by alg-1(-). | 54 | 2.16 | 1.94e-05 |
| Genes down-regulated after 50 um Quercetin treatment. Fold change < 0.8. | 13 | 5.11 | 6.13e-04 |
| Genes enriched in L4 larva intestinal AIN-2 miRISCs, potentially involved in non-development processes. Pges-1-ain-2-gfp IP was performed in synchronized L4 larva. | 22 | 2.92 | 2.35e-03 |
| Genes depleted in muscle cells (24hr muscle dataset). Dissociated myo-3::GFP embryos were cultured for 24 hours before FACS sorting. | 51 | 1.83 | 4.34e-03 |
| Genes with increased expression after 24 hours of infection by P.lumniescens Fold changes shown are pathogen vs OP50. WBPaper00038438:P.lumniescens\_24hr\_upregulated\_RNAseq | 81 | 1.53 | 7.12e-03 |
| Class E gene expression showed no change in lin-14(lf) in L1, up regulation in lin-4(lf) in L2. | 18 | 3.06 | 7.80e-03 |
| Genes with expression enriched in PVD and OLL neurons. Data sets were normalized by RMA and transcripts showing relative PVD enrichment (>= 1.5X) vs. the reference sample were identified by SAM analysis (False Discovery Rate, FDR < 1%). | 61 | 1.60 | 2.70e-02 |
| Genes with increased expression after 24 hours of infection by P.lumniescens Fold changes shown are pathogen vs OP50. WBPaper00038438:P.lumniescens\_24hr\_upregulated\_TilingArray | 93 | 1.40 | 3.57e-02 |
| Genes differentially expressed in control vs after UVC exposure and EtBr treatment at the 3h timepoint (3 h after the third UVC dose (51h), which is also 3 h after being placed on food). | 94 | 1.40 | 3.81e-02 |
| A complete list of the genes that showed differential expression in a slr-2 mutant strain. | 50 | 1.66 | 4.66e-02 |

### Motifs enriched

|  |  |  |  |  |  |
| --- | --- | --- | --- | --- | --- |
| **Motif** | **Logo** | **Possible orthologs** | **Number of motifs in cluster** | **Enrichment** | **FDR corrected p** |
| HepG2\_FOXA2\_HudsonAlpha |  | lin-31 let-381 | 108 | 2.86 | 1.2e-21 |
| Foxa2\_2830 |  | fkh-7 (0.8) lin-31 fkh-10 fkh-8 let-381 daf-16 | 159 | 1.91 | 2.3e-17 |
| pTH9116 |  | pha-4 (0.89) fkh-7 (0.8) lin-31 fkh-8 fkh-10 let-381 daf-16 | 159 | 1.89 | 5.5e-17 |
| V$XFD3\_01 |  | ceh-20 cfi-1 let-381 | 146 | 1.95 | 7.0e-16 |
| V$FREAC7\_01 |  | lin-31 | 145 | 1.91 | 5.9e-15 |
| pTH3043 |  | lin-31 fkh-10 let-381 C34D1.1 | 161 | 1.76 | 2.7e-14 |
| FOXO1\_si |  | fkh-9 (0.68) irx-1 daf-16 | 156 | 1.73 | 6.5e-13 |
| pTH10777 |  | dmd-3 | 153 | 1.71 | 4.7e-12 |
| MA0538.1 |  | daf-12 (0.74) hif-1 Y5F2A.4 ceh-9 ztf-3 | 120 | 1.96 | 6.1e-12 |
| EGR4\_1 |  | klf-2 (0.68) ZC328.2 (0.55) klf-1 | 106 | 2.09 | 1.2e-11 |
| pTH3477 |  | daf-16 | 149 | 1.72 | 1.2e-11 |
| FOXO1\_3 |  | ZC328.2 (0.55) daf-16 | 90 | 2.30 | 1.4e-11 |
| TBX20\_1 |  | mab-9 tbx-39 tbx-38 tbx-43 tbx-42 | 148 | 1.72 | 1.5e-11 |
| pTH2846 |  | lin-31 | 150 | 1.71 | 1.7e-11 |
| ZNF75A\_1 |  | lag-1 F26F4.8 ztf-3 | 140 | 1.77 | 1.9e-11 |
| pTH6591 |  | lin-31 | 156 | 1.65 | 5.0e-11 |
| Bbx\_3753 |  | nhr-208 cfi-1 lim-6 gei-3 | 149 | 1.69 | 5.2e-11 |
| pTH10034 |  | nhr-66 (0.72) | 132 | 1.80 | 5.3e-11 |
| NR4A2\_si |  | nhr-213 (0.88) nhr-71 (0.69) nhr-10 nhr-68 nhr-6 nhr-2 Y67D8A.3 | 124 | 1.86 | 5.4e-11 |
| pTH10807 |  | F13H6.1 (0.66) nhr-177 nhr-86 nhr-2 | 116 | 1.93 | 5.5e-11 |
| pTH9300 |  | dmd-3 C34D1.1 | 117 | 1.92 | 6.7e-11 |
| Hr46\_FlyReg\_FBgn0000448 |  | nhr-213 (0.88) lin-31 | 151 | 1.67 | 6.9e-11 |
| MA0331.1 |  | unc-120 | 135 | 1.77 | 8.6e-11 |
| Sox15\_3457 |  | sox-4 | 127 | 1.83 | 9.2e-11 |
| pTH8330 |  | ztf-6 C34D1.1 gei-11 | 147 | 1.69 | 1.1e-10 |
| pTH6641 |  | lin-31 | 148 | 1.68 | 1.2e-10 |
| Sox21\_3417 |  | sox-4 pop-1 gei-3 C05C9.3 | 131 | 1.79 | 1.3e-10 |
| CG12029\_SANGER\_10\_FBgn0035454 |  | klf-2 (0.68) klf-1 | 108 | 1.98 | 1.3e-10 |
| MA0253.1 |  | ceh-22 (0.95) ceh-24 dsc-1 | 129 | 1.80 | 1.4e-10 |
| Nkx2-9\_3082 |  | ceh-24 dsc-1 | 143 | 1.70 | 1.9e-10 |
| pTH9237 |  | mel-28 | 131 | 1.78 | 2.0e-10 |
| pTH3516 |  | daf-19 | 141 | 1.71 | 2.4e-10 |
| BARHL2\_6 |  | lin-39 (-0.53) ceh-43 ceh-1 ceh-14 ceh-31 | 162 | 1.58 | 3.4e-10 |
| Hth\_SOLEXA\_FBgn0001235 |  | ceh-20 ces-1 ceh-32 F55C5.11 | 118 | 1.86 | 4.2e-10 |
| MA0007.2 |  | nhr-255 npax-1 lin-14 | 147 | 1.66 | 4.3e-10 |
| V$PAX5\_02 |  | pax-3 pax-2 | 137 | 1.71 | 4.8e-10 |
| pTH9108 |  | daf-12 (0.74) nhr-5 | 113 | 1.89 | 5.0e-10 |
| MA0015.1 |  | che-1 K11D2.4 tbp-1 | 126 | 1.79 | 5.1e-10 |
| PURA\_f1 |  | klf-2 (0.68) Y53H1A.2 plp-2 | 144 | 1.67 | 5.4e-10 |
| V$AREB6\_01 |  | ztf-6 tbx-39 | 141 | 1.68 | 6.7e-10 |
| pTH9247 |  | dmd-3 C34D1.1 | 123 | 1.80 | 7.5e-10 |
| pTH9043 |  | sem-2 (0.74) | 182 | 1.48 | 8.8e-10 |
| pTH9174 |  | nhr-28 (0.9) odr-7 lin-1 slr-2 nhr-79 nhr-273 tbx-39 | 146 | 1.64 | 1.0e-09 |
| pTH9215 |  | C34D1.1 | 157 | 1.58 | 1.2e-09 |
| pTH10816 |  | dmd-6 | 194 | 1.43 | 1.2e-09 |
| pTH6497 |  | lin-31 | 96 | 2.03 | 1.3e-09 |
| V$AREB6\_02 |  | ztf-6 | 136 | 1.70 | 1.3e-09 |
| V$CEBP\_01 |  | C48E7.11 | 159 | 1.57 | 1.3e-09 |
| MA0543.1 |  | daf-8 eor-1 | 153 | 1.60 | 1.4e-09 |
| Barhl1\_2 |  | lin-39 (-0.53) ceh-43 lim-7 alr-1 ceh-16 ceh-1 ceh-31 ceh-9 | 135 | 1.70 | 1.5e-09 |
| MA0537.1 |  | blmp-1 | 147 | 1.63 | 1.5e-09 |
| MA0063.1 |  | ceh-19 (0.86) lin-39 (-0.53) ceh-43 lim-7 alr-1 ceh-8 ceh-1 ceh-31 cog-1 ceh-24 ceh-9 ceh-30 | 145 | 1.64 | 1.5e-09 |
| Zfp691\_0895 |  | F21A9.2 CELE\_Y38H8A.5 | 149 | 1.62 | 1.8e-09 |
| FOXB1\_2 |  | hmg-12 (-0.68) ceh-53 (0.67) lin-39 (-0.53) ceh-43 lin-31 let-381 hmbx-1 Y116A8C.22 | 158 | 1.57 | 1.8e-09 |
| Tcf7\_0950 |  | pop-1 | 156 | 1.58 | 1.8e-09 |
| pTH3751 |  | tbx-39 | 102 | 1.95 | 2.0e-09 |
| pTH10640 |  | dmd-5 dmd-4 ceh-32 | 141 | 1.66 | 2.0e-09 |
| pTH9928 |  | fos-1 (0.78) jun-1 sknr-1 crh-1 F45H11.6 | 169 | 1.52 | 2.2e-09 |
| pTH3998 |  | tbx-39 | 143 | 1.64 | 2.2e-09 |
| pTH9925 |  | ztf-11 nhr-100 | 201 | 1.39 | 2.3e-09 |
| TCF4\_2 |  | ztf-6 hlh-2 | 150 | 1.60 | 2.6e-09 |
| F$MCM1\_01 |  | K02D7.2 unc-120 C24A1.2 | 148 | 1.61 | 2.7e-09 |
| pTH9198 |  | dmd-3 | 155 | 1.58 | 2.7e-09 |
| HLH10 |  | unc-120 hlh-10 | 214 | 1.35 | 2.8e-09 |
| pTH9137 |  | nhr-65 (0.87) | 135 | 1.68 | 3.1e-09 |
| pTH10623 |  | scrt-1 | 155 | 1.58 | 3.1e-09 |
| pTH9189 |  | ceh-18 dmd-3 | 183 | 1.45 | 3.3e-09 |
| pTH2820 |  | ZC328.2 (0.55) | 128 | 1.72 | 3.4e-09 |
| Mv104 |  | nhr-62 (0.91) nhr-19 nhr-2 | 103 | 1.92 | 3.8e-09 |
| pTH10811 |  | nhr-216 (0.64) nhr-84 (0.64) nhr-142 | 159 | 1.55 | 3.9e-09 |
| MA0072.1 |  | nhr-213 (0.88) nhr-118 | 139 | 1.65 | 4.1e-09 |
| V$GATA1\_03 |  | elt-1 alr-1 | 150 | 1.59 | 4.6e-09 |
| I$CROC\_01 |  | mef-2 (0.85) mel-28 let-381 Y116A8C.22 Y61A9LA.9 | 114 | 1.82 | 4.7e-09 |
| MA0599.1 |  | klf-2 (0.68) ZC328.2 (0.55) klf-1 | 116 | 1.80 | 5.0e-09 |
| pTH10722 |  | ref-2 eor-1 egrh-3 | 98 | 1.96 | 5.2e-09 |
| Hr51\_SANGER\_5\_FBgn0034012 |  | sox-4 pop-1 nhr-100 | 171 | 1.49 | 6.6e-09 |
| Irx3\_1 |  | irx-1 | 162 | 1.53 | 6.6e-09 |
| pTH10718 |  | egl-43 (0.71) | 150 | 1.58 | 6.9e-09 |
| pTH9097 |  | Y116A8C.22 | 167 | 1.50 | 7.7e-09 |
| pTH9242 |  | mel-28 | 155 | 1.56 | 7.7e-09 |
| CG2052\_SANGER\_2.5\_FBgn0039905 |  | fkh-7 (0.8) lin-29 (0.78) mel-28 | 180 | 1.45 | 8.6e-09 |
| MA0262.1 |  | hsf-1 mab-3 | 163 | 1.52 | 9.6e-09 |
| MA0098.2 |  | lin-1 C24A1.2 | 126 | 1.71 | 9.9e-09 |
| PAX5\_si |  | D1081.8 pax-2 F45H11.6 | 137 | 1.64 | 1.1e-08 |
| YBOX1\_f2 |  | ceh-20 lin-31 dro-1 nfya-2 cey-3 | 127 | 1.70 | 1.2e-08 |
| pTH6445 |  | ceh-5 | 122 | 1.73 | 1.3e-08 |
| pTH9163 |  | nhr-3 (0.7) | 171 | 1.48 | 1.4e-08 |
| KLF8\_f1 |  | ZC328.2 (0.55) klf-1 | 120 | 1.74 | 1.4e-08 |
| pTH6003 |  | nhr-182 (0.81) nhr-134 (0.8) | 128 | 1.69 | 1.4e-08 |
| pTH6164 |  | ceh-2 (0.91) pha-2 (0.76) ceh-45 (0.74) ceh-53 (0.67) lin-39 (-0.53) ceh-43 lim-7 alr-1 ceh-16 ceh-18 ceh-1 lim-4 ceh-12 ceh-36 ceh-14 cog-1 ceh-31 ceh-10 ceh-23 mls-2 and 9 others  [full list] | 127 | 1.69 | 1.4e-08 |
| pTH6327 |  | dsc-1 | 159 | 1.53 | 1.4e-08 |
| SMAD3\_f1 |  | daf-8 | 142 | 1.61 | 1.5e-08 |
| V$GATA3\_02 |  | elt-1 ztf-29 | 154 | 1.55 | 1.6e-08 |
| ZBTB7A\_1 |  | ZC328.2 (0.55) ref-2 lin-1 ztf-14 pax-3 | 137 | 1.63 | 1.6e-08 |
| V$PBX1\_01 |  | lin-39 (-0.53) ceh-20 ceh-12 | 120 | 1.74 | 1.7e-08 |
| EHF\_1 |  | lin-1 nhr-19 C24A1.2 | 108 | 1.83 | 1.7e-08 |
| pTH9182 |  | tbx-39 | 149 | 1.57 | 1.8e-08 |
| pTH9080 |  | mnm-2 | 133 | 1.65 | 1.8e-08 |
| gl\_FlyReg\_FBgn0004618 |  | ces-1 ceh-24 C34H4.5 T22H9.4 | 128 | 1.68 | 2.1e-08 |
| ONECUT3\_1 |  | ceh-48 dsc-1 | 141 | 1.61 | 2.1e-08 |
| V$GATA3\_01 |  | elt-1 | 146 | 1.58 | 2.3e-08 |
| Tcf7l2\_3461 |  | ceh-20 pop-1 | 223 | 1.29 | 2.5e-08 |
| Mv88 |  | mef-2 (0.85) | 167 | 1.48 | 2.7e-08 |
| pTH7032 |  | F52B11.1 | 105 | 1.83 | 2.8e-08 |
| SOX2\_3 |  | grh-1 dmd-4 sox-4 | 155 | 1.53 | 2.9e-08 |
| MA0498.1 |  | lin-39 (-0.53) lin-32 ceh-32 | 138 | 1.61 | 3.0e-08 |
| pTH10717 |  | syd-9 lsy-2 lsl-1 | 124 | 1.69 | 3.2e-08 |
| MA0594.1 |  | lin-39 (-0.53) hbl-1 php-3 | 181 | 1.43 | 3.3e-08 |
| pTH9244 |  | tbx-39 | 136 | 1.62 | 3.3e-08 |
| pTH9709 |  | die-1 (0.62) | 177 | 1.44 | 3.4e-08 |
| pTH6486 |  | nhr-145 | 128 | 1.66 | 3.7e-08 |
| pTH3086 |  | klf-2 (0.68) sptf-3 klf-1 | 104 | 1.83 | 4.0e-08 |
| pTH9076 |  | C01G12.1 | 99 | 1.88 | 4.0e-08 |
| pTH5508 |  | nhr-213 (0.88) nhr-10 nhr-19 nhr-69 nhr-2 | 118 | 1.72 | 4.1e-08 |
| pTH9164 |  | ceh-26 | 145 | 1.57 | 4.2e-08 |
| pTH9125 |  | sox-4 egl-13 K11D2.4 | 138 | 1.60 | 4.6e-08 |
| POU3F1\_1 |  | ceh-18 sox-4 ceh-6 tbp-1 | 171 | 1.46 | 4.6e-08 |
| Elf2 |  | lin-1 F19F10.1 C24A1.2 | 55 | 2.60 | 4.8e-08 |
| GATA4\_f1 |  | elt-1 | 112 | 1.76 | 5.0e-08 |
| pTH9260 |  | mel-28 | 135 | 1.62 | 5.2e-08 |
| V$FOXJ2\_02 |  | elt-1 lin-31 | 164 | 1.48 | 5.2e-08 |
| V$TATA\_01 |  | tbp-1 | 128 | 1.66 | 5.2e-08 |
| pTH9261 |  | lin-48 (0.56) pax-3 dmd-3 | 112 | 1.76 | 5.6e-08 |
| tll\_FlyReg\_FBgn0003720 |  | nhr-62 (0.91) nhr-239 (0.66) nhr-2 | 116 | 1.73 | 5.6e-08 |
| pTH1001 |  | dnj-17 | 93 | 1.92 | 6.0e-08 |
| pTH9135 |  | pop-1 | 136 | 1.60 | 6.7e-08 |
| So\_Cell\_FBgn0003460 |  | ztf-28 ceh-32 skn-1 | 83 | 2.03 | 7.1e-08 |
| Mw160 |  | nhr-68 | 180 | 1.42 | 7.1e-08 |
| V$CETS1P54\_02 |  | C52B9.2 | 107 | 1.79 | 7.2e-08 |
| MA0163.1 |  | C09F5.3 (-0.65) Y53H1A.2 D1081.8 | 113 | 1.74 | 7.2e-08 |
| pTH9254 |  | mel-28 | 147 | 1.55 | 7.3e-08 |
| V$FAC1\_01 |  | gei-8 | 140 | 1.58 | 7.7e-08 |
| Optix\_SOLEXA\_FBgn0025360 |  | ceh-34 (0.71) elt-1 elt-6 egl-27 elt-7 ceh-32 elt-3 | 142 | 1.57 | 8.7e-08 |
| Dlx2\_2273 |  | ceh-43 | 121 | 1.68 | 8.8e-08 |
| Hmx1\_3423 |  | ceh-9 | 158 | 1.50 | 9.4e-08 |
| MA0033.1 |  | hlh-32 lin-31 hlh-15 ngn-1 hlh-8 | 131 | 1.62 | 9.7e-08 |
| pTH8399 |  | lin-54 | 190 | 1.38 | 1.0e-07 |
| Msx2\_3449 |  | eyg-1 (0.77) ceh-45 (0.74) lin-39 (-0.53) alr-1 ceh-1 ceh-10 | 143 | 1.56 | 1.0e-07 |
| pTH9907 |  | nhr-34 (0.6) | 111 | 1.74 | 1.1e-07 |
| V$CART1\_01 |  | lin-39 (-0.53) ceh-43 alr-1 ceh-18 ceh-12 pal-1 | 137 | 1.59 | 1.1e-07 |
| pTH10038 |  | sox-4 F56D1.1 gei-3 | 161 | 1.48 | 1.2e-07 |
| Phox2b\_3948 |  | ceh-45 (0.74) ceh-53 (0.67) lin-39 (-0.53) ceh-43 alr-1 ceh-18 ceh-1 ceh-12 npax-3 | 94 | 1.88 | 1.2e-07 |
| V$CEBPA\_01 |  | C48E7.11 | 99 | 1.83 | 1.3e-07 |
| NR2E3\_f1 |  | lin-39 (-0.53) lin-1 nhr-100 | 132 | 1.61 | 1.3e-07 |
| Nkx1-1\_3856 |  | ceh-30 | 130 | 1.62 | 1.3e-07 |
| pTH2283 |  | odd-2 (0.69) | 128 | 1.63 | 1.4e-07 |
| V$EN1\_01 |  | atf-2 ceh-16 | 122 | 1.66 | 1.5e-07 |
| HLH25 |  | aha-1 lin-22 hlh-27 hlh-28 | 128 | 1.63 | 1.5e-07 |
| MA0547.1 |  | ceh-2 (0.91) skn-1 | 95 | 1.86 | 1.8e-07 |
| V$BRN2\_01 |  | ceh-18 | 181 | 1.40 | 2.0e-07 |
| pTH5561 |  | nhr-239 (0.66) | 138 | 1.57 | 2.0e-07 |
| pTH6408 |  | irx-1 | 91 | 1.89 | 2.2e-07 |
| Barx1\_2877 |  | ceh-43 | 137 | 1.57 | 2.2e-07 |
| Dlx1\_1741 |  | ceh-43 | 149 | 1.52 | 2.2e-07 |
| HepG2\_GABP\_HudsonAlpha |  | lin-1 C24A1.2 | 149 | 1.52 | 2.3e-07 |
| RFX3\_f1 |  | F52B5.7 daf-19 | 121 | 1.65 | 2.6e-07 |
| Hlf\_1 |  | atf-2 ces-2 C48E7.11 Y51H4A.4 C01B12.2 F23F12.9 | 102 | 1.78 | 2.6e-07 |
| Pbx1\_3203 |  | ceh-20 | 165 | 1.45 | 2.7e-07 |
| pTH8983 |  | tag-347 | 119 | 1.66 | 2.8e-07 |
| CG8765\_SANGER\_5\_FBgn0036900 |  | H20J04.3 | 157 | 1.48 | 2.9e-07 |
| Elf4 |  | C24A1.2 | 54 | 2.48 | 3.0e-07 |
| pTH10823 |  | B0310.2 | 127 | 1.61 | 3.3e-07 |
| Tcf3\_3787 |  | pop-1 | 218 | 1.28 | 3.4e-07 |
| pTH8863 |  | hmg-12 (-0.68) | 136 | 1.56 | 3.6e-07 |
| CDC5L\_si |  | hlh-32 irx-1 D1081.8 | 136 | 1.56 | 3.7e-07 |
| CG4854\_SANGER\_10\_FBgn0038766 |  | mxl-1 (-0.69) K11D2.4 | 177 | 1.40 | 3.9e-07 |
| V$HOX13\_01 |  | lin-39 (-0.53) | 149 | 1.50 | 4.1e-07 |
| TBX3\_f1 |  | ceh-45 (0.74) tbx-39 | 106 | 1.73 | 4.4e-07 |
| pTH9913 |  | fos-1 (0.78) skn-1 | 163 | 1.45 | 4.5e-07 |
| pTH5111 |  | mxl-1 (-0.69) mxl-2 (-0.58) hlh-30 (0.56) ref-1 aha-1 lin-22 hlh-26 C27D6.4 | 119 | 1.65 | 4.8e-07 |
| pTH8998 |  | mab-3 daf-19 | 144 | 1.52 | 5.0e-07 |
| pTH5423 |  | klf-2 (0.68) | 71 | 2.09 | 5.1e-07 |
| pTH6449 |  | ceh-43 | 136 | 1.55 | 5.3e-07 |
| Barx2\_3447 |  | ceh-43 | 124 | 1.61 | 5.5e-07 |
| pTH5922 |  | ceh-24 | 82 | 1.94 | 6.7e-07 |
| MA0118.1 |  | ref-2 | 105 | 1.73 | 6.8e-07 |
| pTH2936 |  | nhr-239 (0.66) | 141 | 1.53 | 7.0e-07 |
| pTH9951 |  | mex-6 pal-1 | 126 | 1.60 | 7.0e-07 |
| ALX1\_si |  | alr-1 cfi-1 ceh-14 ZC204.2 | 161 | 1.44 | 7.2e-07 |
| pTH8341 |  | unc-86 ceh-18 | 100 | 1.76 | 7.3e-07 |
| nau\_da\_SANGER\_5\_FBgn0002922 |  | hlh-15 hlh-2 K02D7.2 hlh-1 ces-1 hlh-8 | 150 | 1.49 | 7.5e-07 |
| HXD10\_f1 |  | php-3 nhr-2 | 136 | 1.55 | 7.5e-07 |
| pTH5714 |  | nhr-239 (0.66) | 106 | 1.72 | 7.6e-07 |
| pTH5887 |  | lin-39 (-0.53) | 127 | 1.59 | 7.7e-07 |
| pTH9924 |  | nhr-46 (0.78) | 163 | 1.44 | 7.9e-07 |
| TFEB\_f1 |  | pax-1 (0.92) hlh-30 (0.56) aha-1 mdl-1 | 114 | 1.66 | 8.0e-07 |
| GRHL1\_2 |  | grh-1 | 105 | 1.72 | 8.2e-07 |
| ZN384\_f1 |  | lin-29 (0.78) K11D2.4 | 182 | 1.37 | 8.3e-07 |
| MA0225.1 |  | lin-39 (-0.53) lim-7 unc-86 cfi-1 php-3 | 131 | 1.57 | 8.3e-07 |
| T-47D\_GATA3\_HudsonAlpha |  | elt-1 | 165 | 1.43 | 8.4e-07 |
| Dlx3\_1030 |  | ceh-43 | 145 | 1.50 | 8.6e-07 |
| pTH9044 |  | nhr-177 bed-3 F26F4.8 mbr-1 | 130 | 1.57 | 8.9e-07 |
| pTH5119 |  | cfi-1 | 115 | 1.65 | 9.6e-07 |
| Isl2\_3430 |  | lim-7 alr-1 ceh-18 | 137 | 1.53 | 1.0e-06 |
| pTH1292 |  | pzf-1 ceh-24 | 144 | 1.50 | 1.0e-06 |
| Nsy-7 |  | hmg-5 nsy-7 | 236 | 1.22 | 1.1e-06 |
| pTH6423 |  | pha-2 (0.76) | 97 | 1.77 | 1.1e-06 |
| pTH6589 |  | nhr-213 (0.88) nhr-15 (0.73) nhr-239 (0.66) nhr-2 | 98 | 1.76 | 1.1e-06 |
| V$GR\_Q6 |  | nhr-255 | 160 | 1.44 | 1.1e-06 |
| PTF1A\_f1 |  | lin-32 hlh-2 | 137 | 1.53 | 1.1e-06 |
| ETS2\_f1 |  | lin-1 C24A1.2 | 171 | 1.40 | 1.1e-06 |
| pTH3041 |  | atf-2 | 98 | 1.76 | 1.2e-06 |
| pTH5078 |  | ces-2 | 112 | 1.66 | 1.2e-06 |
| CENPB\_1 |  | F21D5.4 F52B11.1 | 137 | 1.53 | 1.2e-06 |
| FLI1\_f1 |  | lin-1 | 126 | 1.58 | 1.2e-06 |
| FOXB1\_1 |  | nhr-68 lin-31 F55C5.11 | 153 | 1.46 | 1.3e-06 |
| pTH9900 |  | C46E10.8 C33G8.2 | 164 | 1.42 | 1.4e-06 |
| pTH5781 |  | ceh-32 | 129 | 1.56 | 1.4e-06 |
| Atf1\_3026 |  | crh-1 | 136 | 1.53 | 1.5e-06 |
| Elf3 |  | C24A1.2 | 109 | 1.67 | 1.5e-06 |
| Elf3\_3876 |  | C24A1.2 | 117 | 1.63 | 1.5e-06 |
| MA0032.1 |  | let-381 | 198 | 1.32 | 1.5e-06 |
| pTH6508 |  | nhr-36 | 145 | 1.49 | 1.5e-06 |
| Cdx2\_4272 |  | ceh-13 | 140 | 1.51 | 1.6e-06 |
| MA0038.1 |  | odd-1 lim-6 | 174 | 1.39 | 1.6e-06 |
| pTH9082 |  | mab-23 | 91 | 1.81 | 1.6e-06 |
| OTX2\_1 |  | pha-2 (0.76) ceh-45 (0.74) dve-1 (0.71) ceh-53 (0.67) alr-1 ceh-36 | 146 | 1.49 | 1.6e-06 |
| pTH5270 |  | hlh-32 hlh-16 ngn-1 | 127 | 1.57 | 1.8e-06 |
| pTH9155 |  | lin-48 (0.56) D1081.8 B0310.2 | 114 | 1.64 | 1.8e-06 |
| Hoxb5\_3122 |  | lin-39 (-0.53) | 115 | 1.63 | 1.9e-06 |
| Nkx1-2\_3214 |  | ceh-30 | 120 | 1.60 | 2.0e-06 |
| V$OCT1\_06 |  | ceh-18 ztf-9 | 129 | 1.55 | 2.1e-06 |
| Hmbox1\_2674 |  | hmbx-1 | 118 | 1.61 | 2.1e-06 |
| CEBPE\_f1 |  | C48E7.11 R07H5.10 | 161 | 1.42 | 2.1e-06 |
| Abd-B\_FlyReg\_FBgn0000015 |  | lin-39 (-0.53) ceh-13 ceh-24 php-3 T27F2.4 D1005.3 | 124 | 1.58 | 2.3e-06 |
| pTH10645 |  | nhr-7 (0.88) elt-1 nhr-10 nhr-100 | 179 | 1.36 | 2.3e-06 |
| pTH10647 |  | nhr-232 | 165 | 1.41 | 2.3e-06 |
| pTH9335 |  | mel-28 | 126 | 1.57 | 2.3e-06 |
| Vsx1\_1728 |  | alr-1 | 171 | 1.39 | 2.5e-06 |
| Hlxb9\_3422 |  | ceh-12 | 133 | 1.53 | 2.5e-06 |
| EN1\_2 |  | ceh-2 (0.91) ceh-16 | 132 | 1.53 | 2.5e-06 |
| V$OCT1\_03 |  | ceh-18 | 116 | 1.61 | 2.7e-06 |
| BARHL2\_1 |  | ceh-31 | 122 | 1.58 | 2.9e-06 |
| V$PAX2\_02 |  | pax-1 (0.92) | 151 | 1.45 | 3.0e-06 |
| ECC-1\_ERALPHA\_HudsonAlpha |  | nhr-71 (0.69) nhr-6 nhr-43 | 106 | 1.67 | 3.0e-06 |
| I$KR\_01 |  | ZK177.3 B0310.2 | 128 | 1.55 | 3.2e-06 |
| pTH8333 |  | F39B2.1 ZC416.1 | 85 | 1.83 | 3.4e-06 |
| I$DFD\_01 |  | lin-39 (-0.53) | 109 | 1.65 | 3.5e-06 |
| pTH9177 |  | F10B5.3 hsf-1 Y53C10A.3 | 127 | 1.55 | 3.5e-06 |
| Emx2\_3420 |  | ceh-2 (0.91) | 230 | 1.22 | 3.9e-06 |
| pTH8216 |  | Y116A8C.22 | 138 | 1.50 | 4.0e-06 |
| Hoxc4\_3491 |  | lin-39 (-0.53) | 117 | 1.60 | 4.0e-06 |
| pTH9165 |  | ztf-27 (0.67) | 125 | 1.55 | 4.0e-06 |
| Hoxa5\_3415 |  | lin-39 (-0.53) | 109 | 1.64 | 4.1e-06 |
| pTH3064 |  | crh-1 | 140 | 1.49 | 4.1e-06 |
| Gsh2\_3990 |  | ceh-31 | 113 | 1.62 | 4.1e-06 |
| Spt15 |  | tbp-1 | 129 | 1.54 | 4.2e-06 |
| pTH9384 |  | cfi-1 | 75 | 1.93 | 4.2e-06 |
| rn\_SOLEXA\_5\_FBgn0259172 |  | lin-29 (0.78) | 193 | 1.31 | 4.3e-06 |
| MA0124.1 |  | ceh-48 ceh-24 | 136 | 1.50 | 4.4e-06 |
| pTH6562 |  | ceh-5 | 134 | 1.51 | 4.5e-06 |
| GATA6\_f2 |  | elt-1 end-3 | 160 | 1.41 | 4.5e-06 |
| MA0139.1 |  | Y5F2A.4 F58G1.2 | 131 | 1.52 | 5.0e-06 |
| Hey\_SANGER\_5\_FBgn0027788 |  | lin-22 gei-11 | 134 | 1.51 | 5.3e-06 |
| Sox17\_2837 |  | sox-4 | 190 | 1.32 | 5.6e-06 |
| Spdef |  | lin-1 | 98 | 1.70 | 5.8e-06 |
| MA0454.1 |  | odd-2 (0.69) odd-1 | 216 | 1.25 | 6.0e-06 |
| pTH5080 |  | fos-1 (0.78) atf-5 crh-1 | 127 | 1.53 | 6.3e-06 |
| Arid3a\_3875 |  | cfi-1 | 128 | 1.53 | 6.7e-06 |
| MA0161.1 |  | nfi-1 F49E12.6 | 128 | 1.53 | 6.8e-06 |
| Pou3f3\_3235 |  | ceh-6 | 125 | 1.54 | 7.0e-06 |
| Lhx1\_2240 |  | lim-7 | 148 | 1.44 | 7.0e-06 |
| V$MYB\_Q6 |  | D1081.8 | 87 | 1.78 | 7.1e-06 |
| MA0470.1 |  | efl-1 F49E12.6 | 91 | 1.75 | 7.3e-06 |
| pTH6636 |  | egl-5 | 121 | 1.56 | 7.4e-06 |
| pTH10028 |  | nhr-204 (0.56) | 146 | 1.45 | 7.4e-06 |
| pTH8991 |  | cey-3 | 217 | 1.24 | 7.9e-06 |
| Mafk\_3106 |  | F45H11.6 | 114 | 1.59 | 8.1e-06 |
| pTH10013 |  | nhr-168 | 195 | 1.30 | 8.4e-06 |
| MA0027.1 |  | ceh-16 | 121 | 1.55 | 8.7e-06 |
| pTH10630 |  | lsy-27 | 240 | 1.19 | 8.7e-06 |
| Irx2\_0900 |  | irx-1 | 113 | 1.59 | 8.8e-06 |
| pTH9969 |  | pag-3 | 267 | 1.11 | 8.8e-06 |
| Elf5 |  | C24A1.2 | 116 | 1.58 | 8.8e-06 |
| pTH8649 |  | mbr-1 | 99 | 1.68 | 8.8e-06 |
| pTH5118 |  | cfi-1 | 147 | 1.44 | 9.0e-06 |
| pTH2684 |  | fos-1 (0.78) | 141 | 1.47 | 9.0e-06 |
| gl\_SANGER\_5\_FBgn0004618 |  | ztf-28 che-1 ces-1 | 116 | 1.58 | 9.2e-06 |
| MAFA\_f1 |  | daf-8 pax-2 F45H11.6 | 150 | 1.43 | 9.5e-06 |
| pTH5250 |  | C48E7.11 | 107 | 1.63 | 9.5e-06 |
| pTH9934 |  | Y53H1A.2 | 114 | 1.59 | 9.7e-06 |
| Bsx\_3483 |  | ceh-31 | 129 | 1.51 | 1.0e-05 |
| Hoxd9\_2 |  | lin-39 (-0.53) ceh-24 php-3 pal-1 | 165 | 1.38 | 1.1e-05 |
| pTH10654 |  | ceh-90 lsy-27 | 111 | 1.60 | 1.1e-05 |
| pTH9326 |  | nhr-122 | 154 | 1.41 | 1.2e-05 |
| pTH5924 |  | nhr-255 | 145 | 1.44 | 1.3e-05 |
| pTH8985 |  | athp-1 | 137 | 1.47 | 1.3e-05 |
| Six4\_2860 |  | ceh-32 | 95 | 1.69 | 1.4e-05 |
| pTH9380 |  | mel-28 | 147 | 1.43 | 1.4e-05 |
| Vax1\_3499 |  | C02F12.10 | 241 | 1.18 | 1.4e-05 |
| pTH6436 |  | ceh-53 (0.67) | 229 | 1.21 | 1.5e-05 |
| MA0488.1 |  | crh-1 | 104 | 1.63 | 1.5e-05 |
| V$IK2\_01 |  | F26F4.8 | 139 | 1.46 | 1.7e-05 |
| Hoxa4\_3426 |  | lin-39 (-0.53) | 246 | 1.17 | 1.8e-05 |
| pTH5976 |  | mxl-1 (-0.69) hlh-30 (0.56) irx-1 | 141 | 1.45 | 1.9e-05 |
| Spdef\_0905 |  | lin-1 | 126 | 1.51 | 2.0e-05 |
| HeLa-S3\_ZNF274\_UCD |  | C28G1.4 | 103 | 1.62 | 2.0e-05 |
| Etv3 |  | lin-1 | 174 | 1.34 | 2.1e-05 |
| MCR\_f1 |  | nhr-255 | 134 | 1.47 | 2.3e-05 |
| Nkx6-1\_2825 |  | cog-1 | 125 | 1.51 | 2.3e-05 |
| V$TAXCREB\_02 |  | zip-3 crh-1 | 128 | 1.49 | 2.5e-05 |
| V$GATA1\_02 |  | elt-1 | 123 | 1.51 | 2.5e-05 |
| V$AP4\_01 |  | lin-32 hlh-11 hlh-14 hlh-15 hlh-1 | 132 | 1.48 | 2.5e-05 |
| pTH8679 |  | pax-2 | 87 | 1.73 | 2.5e-05 |
| MA0540.1 |  | dpy-27 Y44A6D.3 | 121 | 1.52 | 2.5e-05 |
| V$AHR\_01 |  | aha-1 ahr-1 | 119 | 1.53 | 2.7e-05 |
| Hnf4a\_2640 |  | nhr-62 (0.91) | 113 | 1.56 | 2.8e-05 |
| CUX1\_2 |  | ceh-48 | 144 | 1.43 | 2.8e-05 |
| Pou3f4\_3773 |  | ceh-6 | 154 | 1.39 | 3.2e-05 |
| V$S8\_01 |  | ceh-45 (0.74) | 202 | 1.26 | 3.3e-05 |
| pTH5437 |  | ceh-34 (0.71) | 120 | 1.52 | 3.3e-05 |
| pTH9708 |  | ceh-34 (0.71) | 100 | 1.62 | 3.4e-05 |
| pTH6447 |  | ceh-19 (0.86) | 141 | 1.43 | 3.5e-05 |
| Mf28 |  | elt-1 | 130 | 1.47 | 3.6e-05 |
| En2\_0952 |  | lim-7 ceh-16 ceh-24 lim-6 | 124 | 1.50 | 3.7e-05 |
| Srf\_3509 |  | unc-120 | 129 | 1.48 | 3.7e-05 |
| Six3\_1732 |  | ceh-34 (0.71) | 110 | 1.56 | 3.8e-05 |
| pTH6569 |  | ceh-43 | 127 | 1.48 | 3.9e-05 |
| pTH5257 |  | C48E7.11 | 125 | 1.49 | 4.0e-05 |
| pTH9049 |  | ztf-6 ztf-2 | 46 | 2.29 | 4.1e-05 |
| V$CREB\_01 |  | crh-1 attf-1 | 94 | 1.65 | 4.2e-05 |
| V$TCF11\_01 |  | skn-1 | 148 | 1.40 | 4.4e-05 |
| V$CDXA\_01 |  | ceh-13 php-3 | 207 | 1.25 | 4.5e-05 |
| Pou2f3\_3986 |  | ceh-18 | 165 | 1.35 | 4.6e-05 |
| Hoxb4\_2627 |  | lin-39 (-0.53) | 246 | 1.16 | 4.7e-05 |
| pTH9220 |  | mbr-1 | 148 | 1.40 | 4.8e-05 |
| Vax2\_3500 |  | C02F12.10 | 237 | 1.18 | 5.0e-05 |
| En1\_3123 |  | ceh-53 (0.67) ceh-16 | 125 | 1.48 | 5.1e-05 |
| pTH3819 |  | ceh-18 | 120 | 1.51 | 5.1e-05 |
| pTH9089 |  | ref-2 T22C8.4 | 113 | 1.54 | 5.1e-05 |
| K562\_ZBTB7A\_HudsonAlpha |  | ZC328.2 (0.55) | 100 | 1.60 | 5.6e-05 |
| pTH8411 |  | tbx-39 | 150 | 1.39 | 6.0e-05 |
| Tcf1\_2666 |  | hmbx-1 | 107 | 1.56 | 6.0e-05 |
| pTH9387 |  | C34D1.1 | 88 | 1.68 | 6.3e-05 |
| pTH10798 |  | Y75B8A.6 | 88 | 1.68 | 6.6e-05 |
| V$NCX\_01 |  | ceh-19 (0.86) | 81 | 1.73 | 6.8e-05 |
| Hoxa10\_2318 |  | ceh-24 | 132 | 1.45 | 7.2e-05 |
| ARNT2\_si |  | aha-1 C46E10.9 | 128 | 1.46 | 7.7e-05 |
| pTH9216 |  | ceh-18 | 158 | 1.36 | 8.2e-05 |
| Hoxb9\_3413 |  | ceh-24 pal-1 | 147 | 1.39 | 8.5e-05 |
| Pknox2\_3077 |  | ceh-32 | 224 | 1.20 | 8.9e-05 |
| Hoxd1\_3448 |  | ceh-12 | 226 | 1.19 | 9.9e-05 |
| Six1\_0935 |  | ceh-32 | 85 | 1.68 | 1.0e-04 |
| pTH10041 |  | ztf-29 | 135 | 1.43 | 1.1e-04 |
| pTH8996 |  | daf-8 sma-4 hlh-8 | 146 | 1.39 | 1.1e-04 |
| pTH9245 |  | ceh-18 | 136 | 1.42 | 1.1e-04 |
| Smad3\_3805 |  | daf-8 | 228 | 1.19 | 1.1e-04 |
| pTH9353 |  | ceh-51 | 132 | 1.44 | 1.1e-04 |
| Evx1\_3952 |  | ceh-53 (0.67) | 122 | 1.47 | 1.2e-04 |
| pTH9297 |  | ceh-18 | 138 | 1.41 | 1.2e-04 |
| pTH3046 |  | Y116A8C.22 | 146 | 1.39 | 1.2e-04 |
| pTH3997 |  | C04F5.9 | 201 | 1.25 | 1.2e-04 |
| pTH8556 |  | pax-2 | 110 | 1.52 | 1.3e-04 |
| Hoxa7\_3750 |  | lin-39 (-0.53) | 244 | 1.15 | 1.3e-04 |
| pTH6268 |  | ceh-2 (0.91) | 212 | 1.22 | 1.3e-04 |
| Meox1\_2310 |  | ceh-31 | 118 | 1.48 | 1.4e-04 |
| pTH1049 |  | elt-1 | 141 | 1.40 | 1.4e-04 |
| Hoxc8\_3429 |  | lin-39 (-0.53) | 88 | 1.64 | 1.6e-04 |
| Hoxa3\_2783 |  | lin-39 (-0.53) | 234 | 1.17 | 1.6e-04 |
| Irx5\_2385 |  | irx-1 | 112 | 1.50 | 1.6e-04 |
| Hoxa6\_1040 |  | lin-39 (-0.53) | 111 | 1.51 | 1.7e-04 |
| V$GATA1\_01 |  | elt-1 | 111 | 1.51 | 1.7e-04 |
| I$UBX\_01 |  | lin-39 (-0.53) | 214 | 1.21 | 1.7e-04 |
| pTH10031 |  | mbr-1 | 93 | 1.60 | 1.7e-04 |
| HES1\_f1 |  | lin-22 | 128 | 1.44 | 1.8e-04 |
| Sox1\_2631 |  | sox-4 | 169 | 1.31 | 1.9e-04 |
| srp\_FlyReg\_FBgn0003507 |  | lin-39 (-0.53) elt-1 | 206 | 1.23 | 1.9e-04 |
| Pou2f1\_3081 |  | ceh-18 | 120 | 1.46 | 2.0e-04 |
| MA0095.2 |  | lsy-2 | 165 | 1.32 | 2.0e-04 |
| Nkx6-3\_3446 |  | cog-1 | 176 | 1.29 | 2.1e-04 |
| Pou3f2\_2824 |  | lin-39 (-0.53) ceh-18 ceh-6 | 172 | 1.30 | 2.1e-04 |
| pTH5690 |  | ceh-32 | 115 | 1.48 | 2.1e-04 |
| Oli\_da\_SANGER\_5\_2\_FBgn0032651 |  | hlh-32 hlh-12 hlh-15 ngn-1 hlh-8 | 122 | 1.45 | 2.4e-04 |
| pTH7875 |  | mel-28 | 126 | 1.43 | 2.5e-04 |
| pTH9314 |  | fos-1 (0.78) atf-6 crh-1 atf-7 C27D6.4 | 218 | 1.20 | 2.5e-04 |
| pTH10769 |  | Y48G1C.6 | 126 | 1.43 | 2.6e-04 |
| Hoxb3\_1720 |  | lin-39 (-0.53) | 237 | 1.16 | 2.6e-04 |
| pTH10808 |  | ztf-19 | 131 | 1.41 | 2.7e-04 |
| Hoxb8\_3780 |  | lin-39 (-0.53) | 83 | 1.65 | 2.9e-04 |
| pTH6482 |  | ceh-19 (0.86) | 105 | 1.52 | 3.0e-04 |
| pTH10027 |  | M03D4.4 | 66 | 1.79 | 3.4e-04 |
| MA0146.2 |  | F58G1.2 aptf-1 | 84 | 1.63 | 3.5e-04 |
| pTH9219 |  | xbp-1 C01B12.2 | 189 | 1.25 | 3.8e-04 |
| pTH10772 |  | ceh-52 | 230 | 1.17 | 3.8e-04 |
| pTH4325 |  | ceh-18 | 145 | 1.36 | 4.3e-04 |
| Hoxa7\_2668 |  | lin-39 (-0.53) | 82 | 1.63 | 4.7e-04 |
| pTH2933 |  | F58G1.2 | 52 | 1.95 | 4.8e-04 |
| TLX1\_f1 |  | ceh-19 (0.86) | 120 | 1.44 | 4.9e-04 |
| Gata5\_3768 |  | elt-1 | 96 | 1.54 | 5.2e-04 |
| Tcf1\_2666 |  | hmbx-1 | 103 | 1.50 | 5.3e-04 |
| pTH9381 |  | ceh-18 | 129 | 1.40 | 5.5e-04 |
| V$GATA6\_01 |  | elt-1 | 144 | 1.35 | 5.6e-04 |
| Lbx2\_3869 |  | mls-2 | 217 | 1.19 | 6.2e-04 |
| pTH5812 |  | ceh-14 | 223 | 1.18 | 6.2e-04 |
| pTH9256 |  | ceh-18 | 134 | 1.38 | 6.3e-04 |
| Hoxa9\_2622 |  | lin-39 (-0.53) php-3 | 144 | 1.35 | 6.4e-04 |
| Poxm\_SOLEXA\_5\_FBgn0003129 |  | pax-2 | 205 | 1.21 | 6.5e-04 |
| pTH8982 |  | ceh-48 | 94 | 1.54 | 6.8e-04 |
| Zfp161\_2858 |  | pzf-1 | 38 | 2.21 | 7.5e-04 |
| Cdx1\_2245 |  | ceh-13 | 126 | 1.40 | 8.0e-04 |
| V$YY1\_01 |  | lsy-2 | 183 | 1.25 | 8.2e-04 |
| pTH9085 |  | nhr-42 (0.83) | 102 | 1.49 | 9.1e-04 |
| pTH5778 |  | egl-5 | 117 | 1.43 | 9.2e-04 |
| pTH9880 |  | end-1 | 123 | 1.40 | 9.7e-04 |
| pTH6106 |  | nhr-182 (0.81) | 119 | 1.42 | 1.0e-03 |
| pTH2280 |  | mnm-2 | 102 | 1.48 | 1.1e-03 |
| pTH10650 |  | nhr-153 (0.85) | 99 | 1.49 | 1.1e-03 |
| ERG\_4 |  | lin-1 | 116 | 1.42 | 1.2e-03 |
| pTH9096 |  | T07C12.11 | 186 | 1.24 | 1.2e-03 |
| Irx3\_0920 |  | irx-1 | 100 | 1.48 | 1.3e-03 |
| Hoxa2\_3079 |  | lin-39 (-0.53) | 234 | 1.15 | 1.4e-03 |
| pTH5914 |  | attf-1 | 94 | 1.50 | 1.6e-03 |
| pTH1294 |  | mel-28 | 116 | 1.41 | 1.7e-03 |
| pTH8745 |  | attf-1 | 175 | 1.25 | 1.7e-03 |
| pTH8318 |  | attf-1 | 179 | 1.25 | 1.7e-03 |
| pTH8671 |  | attf-1 C01B12.2 | 178 | 1.25 | 1.7e-03 |
| Plagl1\_0972 |  | Y53H1A.2 | 79 | 1.59 | 1.7e-03 |
| pTH5891 |  | nhr-49 (0.81) | 103 | 1.46 | 1.7e-03 |
| Pou3f1\_3819 |  | ceh-6 | 117 | 1.40 | 1.8e-03 |
| Cutl1\_3494 |  | ceh-44 | 48 | 1.90 | 1.8e-03 |
| pTH9026 |  | attf-1 | 93 | 1.50 | 1.9e-03 |
| NHLH1\_1 |  | hlh-15 hlh-1 | 118 | 1.39 | 2.2e-03 |
| pTH10788 |  | tbx-33 | 181 | 1.24 | 2.2e-03 |
| Six6\_2267 |  | ceh-34 (0.71) ceh-32 | 91 | 1.50 | 2.2e-03 |
| pTH5928 |  | ceh-34 (0.71) | 82 | 1.55 | 2.3e-03 |
| pTH6478 |  | lim-7 | 77 | 1.58 | 2.5e-03 |
| Hoxa11\_2218 |  | php-3 | 138 | 1.33 | 2.5e-03 |
| pTH10805 |  | ztf-16 (0.92) | 63 | 1.68 | 2.8e-03 |
| Gata3\_1024 |  | elt-1 | 137 | 1.33 | 3.2e-03 |
| HIF1A\_si |  | hif-1 | 97 | 1.45 | 3.9e-03 |
| Gmeb1\_1745 |  | attf-1 | 96 | 1.44 | 5.0e-03 |
| pTH5919 |  | irx-1 | 66 | 1.60 | 6.1e-03 |
| HepG2\_HSF1\_Stanford |  | Y53C10A.3 | 116 | 1.36 | 7.0e-03 |
| pTH10768 |  | med-2 | 197 | 1.18 | 9.7e-03 |
| pTH1014 |  | atf-5 | 62 | 1.57 | 1.6e-02 |
| pTH9149 |  | ztf-30 | 67 | 1.51 | 2.1e-02 |
| Hoxd13\_2356 |  | pal-1 | 157 | 1.22 | 2.6e-02 |
| YY1\_1 |  | lsy-2 | 127 | 1.28 | 2.8e-02 |
| pTH5916 |  | efl-2 | 68 | 1.47 | 3.5e-02 |
| pTH9173 |  | efl-2 | 96 | 1.34 | 4.1e-02 |

### Correlated (and anti-correlated) transcription factors

|  |  |
| --- | --- |
| **Transcription factor** | **Correlation** |
| ceh-22 | 0.95 |
| gmeb-2 | 0.95 |
| nhr-54 | 0.94 |
| cky-1 | 0.94 |
| nhr-130 | 0.93 |
| nhr-203 | 0.92 |
| ztf-16 | 0.92 |
| pax-1 | 0.92 |
| ets-4 | 0.92 |
| nhr-117 | 0.91 |
| nhr-62 | 0.91 |
| ceh-2 | 0.91 |
| nhr-28 | 0.90 |
| nhr-103 | 0.90 |
| nhr-123 | 0.90 |
| peb-1 | 0.89 |
| pha-4 | 0.89 |
| nhr-238 | 0.89 |
| nhr-213 | 0.88 |
| nhr-212 | 0.88 |
| nhr-56 | 0.88 |
| nhr-7 | 0.88 |
| nhr-133 | 0.87 |
| nhr-65 | 0.87 |
| nhr-243 | 0.87 |
| ztf-4 | -0.47 |
| ceh-20 | -0.47 |
| unc-3 | -0.48 |
| Y53H1A.2 | -0.49 |
| ceh-13 | -0.49 |
| elt-1 | -0.49 |
| cey-2 | -0.52 |
| ccch-3 | -0.52 |
| mab-5 | -0.53 |
| lin-39 | -0.53 |
| spe-44 | -0.54 |
| ceh-41 | -0.54 |
| tbx-31 | -0.55 |
| bar-1 | -0.56 |
| cebp-2 | -0.57 |
| mxl-2 | -0.58 |
| him-8 | -0.58 |
| repo-1 | -0.59 |
| Y56A3A.18 | -0.59 |
| C01F6.9 | -0.61 |
| C09F5.3 | -0.65 |
| hmg-11 | -0.67 |
| hmg-12 | -0.68 |
| mxl-1 | -0.69 |
| F21D5.9 | -0.74 |

### ChIP peaks enriched

|  |  |  |  |  |
| --- | --- | --- | --- | --- |
| **Gene** | **Experiment** | **Number of upstream peaks** | **Enrichment** | **FDR corrected p** |
| pha-4 | PHA-4\_Larvae-L2-stage | 80 | 2.81 | 1.1e-15 |
| pha-4 | PHA-4\_Larvae-L4-stage | 84 | 2.14 | 4.0e-10 |
| nhr-77 | NHR-77\_Fed-L1-stage-larvae | 50 | 2.20 | 7.0e-06 |
| fos-1 | FOS-1\_Larvae-L2-stage | 54 | 1.86 | 2.7e-04 |
| nhr-28 | NHR-28\_Larvae-L4-stage | 57 | 1.80 | 3.7e-04 |
| pha-4 | PHA-4\_Young-adult | 24 | 2.51 | 1.7e-03 |
| hpl-2 | HPL-2\_Fed-L1-stage-larvae | 9 | 3.88 | 2.0e-02 |
| elt-3 | ELT-3\_Embryos | 30 | 1.86 | 2.7e-02 |
| sax-3 | SAX-3\_Larvae-L4-stage | 48 | 1.58 | 3.1e-02 |
| aly-2 | ALY-2\_Larvae-L2-stage | 14 | 2.61 | 3.6e-02 |
| nhr-77 | NHR-77\_Larvae-L4-stage | 45 | 1.58 | 4.6e-02 |
